# LincRNA01703 Facilitates CD81^+^ Exosome Secretion to Inhibit Lung Adenocarcinoma Metastasis via the Rab27a/SYTL1/CD81 Complex

**DOI:** 10.3390/cancers15245781

**Published:** 2023-12-09

**Authors:** Yun Huang, Shan Guo, Ying Lin, Liyun Huo, Hongmei Yan, Zhanwen Lin, Zishuo Chen, Junchao Cai, Jueheng Wu, Jie Yuan, Hongyu Guan, Guoyong Wu, Weibin Wu, Tianyu Tao

**Affiliations:** 1 Precision Medicine Institute, The First Affiliated Hospital, Sun Yat-sen University, Guangzhou 510000, China; 2 Advanced Medical Technology Center, The First Affiliated Hospital, Zhongshan School of Medicine, Sun Yat-sen University, Guangzhou 510000, China; 3 Department of Medical Ultrasonics, The First Affiliated Hospital, Sun Yat-sen University, Guangzhou 510000, China; guosh23@mail.sysu.edu.cn; 4 Department of Immunology, Zhongshan School of Medicine, Sun Yat-sen University, Guangzhou 510000, China; 5 Department of Vascular Surgery, The First Affiliated Hospital, Sun Yat-sen University, Guangzhou 510000, China; 6 School of Basic Medical Sciences, Southern Medical University, Guangzhou 510000, China; 7 Department of Microbiology, Zhongshan School of Medicine, Sun Yat-sen University, Guangzhou 510000, China; 8 Department of Biochemistry, Zhongshan School of Medicine, Sun Yat-sen University, Guangzhou 510000, China; 9 Department of Endocrinology and Diabetes Center, The First Affiliated Hospital, Sun Yat-sen University, Guangzhou 510000, China; 10 Department of Thoracic Surgery, The First Affiliated Hospital, Sun Yat-sen University, Guangzhou 510000, China; 11 Department of Cardiothoracic Surgery, The Third Affiliated Hospitalof Sun Yat-sen University, Guangzhou 510000, China

**Keywords:** exosomes, long noncoding RNA, metastasis, lung cancer, CD81

## Abstract

**Simple Summary:**

Metastasis, the hallmark of cancer, is accountable for about 90% of cancer-related deaths. Long noncoding RNA (lncRNA), a subset of the noncoding RNAs, has exhibited involvement in various processes, including immune evasion, cell proliferation, migration, and invasion, across multiple human diseases, notably cancer. The objective of our study was to identify a metastasis-associated lncRNA in lung cancer and elucidate the underlying mechanisms. Our findings indicate that Linc01703, which is notably downregulated in metastatic lung cancer cells, effectively suppresses lung cancer metastasis in vivo. Interestingly, Linc01703 does not directly impact the proliferation and invasion capabilities of lung cancer cells but rather inhibits cancer metastasis by promoting the secretion of CD81^+^ exosomes through the Rab27a/SYTL1/CD81 transport complexes. Consequently, our observations provide new insights into the potential clinical application of CD81^+^ exosome-based cancer therapy.

**Abstract:**

Metastasis, a major cause of cancer-related mortality worldwide, frequently occurs early in the diagnosis of lung adenocarcinoma (LUAD). However, the precise molecular mechanisms governing the aggressive metastatic behavior of LUAD remain incompletely understood. In this study, we present compelling evidence indicating that the long noncoding RNA linc01703 is significantly downregulated in metastatic lung cancer cells. Intriguingly, in vivo experiments revealed that Linc01703 exerted a profound inhibitory effect on lung cancer metastasis without discernible impact on the in vitro proliferation or invasion capacities of LUAD cells. Mechanistically, Linc01703 enhanced the interaction between Rab27a, SYTL1, and CD81, consequently promoting the secretion of CD81^+^ exosomes. These exosomes, in turn, suppressed the infiltration of immune cells within the tumor microenvironment, thereby impeding LUAD metastasis. Importantly, our analysis of lung cancer tissues revealed a correlation between reduced CD81 expression and an unfavorable patient prognosis. Collectively, our findings suggest that Linc01703 functions as a metastasis suppressor by facilitating the secretion of CD81^+^ exosomes through the formation of the Rab27a/SYTL1/CD81 complex.

## 1. Introduction

Lung cancer is the leading cause of cancer-related mortality and one of the most generally diagnosed cancers in the world [1]. Lung adenocarcinoma (LUAD) and lung squamous cell carcinoma (LUSC) are the two main subtypes of non-small cell lung cancer (NSCLC), which account for approximately 85% of all lung cancer cases. Metastasis is the primary cause of death among LUAD patients [2], with approximately 70% of patients being diagnosed at an advanced stage with local or distant metastasis [3,4]. The patients with distant metastasis have a survival time of only about 8 months [3,4]. Despite some clinical advancements, the survival time and quality of life for LUAD patients remain poor [5,6]. Therefore, understanding the mechanisms underlying LUAD metastasis and exploring novel clinical treatment methods are of utmost importance.

The tumor microenvironment (TME) consists of various cellular and acellular components that significantly influence tumorigenesis, metastasis, and clinical outcome [7,8]. Exosomes, extracellular vesicles (EVs) with an average diameter of 100 nm, play a crucial role in intercellular communication by transporting proteins, lipids, and nucleic acids [9]. Tumor-derived exosomes, in particular, have been implicated in the initiation and progression of different cancer processes, including tumor metastasis [10]. The generation of exosomes involves intricate intracellular trafficking steps, such as inward budding of the plasma membrane to form early endosomes, which then give rise to late endosomes and multivesicular bodies (MVBs) that fuse with the plasma membrane for exosome release [11]. Rab family members, particularly Rab27a and Rab27b, which are RAS-related protein (RAB) GTPases, are essential for exosome secretion [11,12]. Rab27a is involved in the docking of exosomes and the rearrangement of the submembrane actin cytoskeleton, while Rab27b regulates the movement of exosomes toward the plasma membrane [12,13]. However, the specific exosome subtype involved in lung cancer metastasis and how to target these exosomes to suppress tumor metastasis remain unknown.

Several tetraspanin (TSPN) family members, including CD9, CD81, and CD63, are major constituents of exosomes and serve as canonical markers [14]. CD81, in particular, is tightly regulated in physiological and pathological processes, including inflammation, pathogen infection, cell adhesion, and tumor development [15,16,17,18]. Interestingly, decreased CD81 expression has been associated with increased metastatic capacity in liver cancer and bladder cancers [19,20], while the knockdown of CD81 has been shown to decrease cell motility and metastasis in melanoma and breast cancer [21,22,23]. These findings suggest that CD81 expression plays a crucial role in tumor development and may serve as a prognostic target for tumor therapy. However, it remains unclear whether CD81 is transmitted to target cells through exosomes and then exerts tumor-promoting or tumor-inhibiting effects in lung cancer.

Long noncoding RNAs (lncRNAs), a newly discovered class of noncoding RNAs, are commonly defined as RNA molecules that are more than 200 nucleotides in length [24] and have been shown to play essential roles in exosome generation, secretion, and cancer metastasis [25,26,27]. In this study, we focus on a lncRNA called LincRNA01703 (Linc01703), which is significantly suppressed in metastatic LUAD cells. However, the functions and downstream mechanisms of Linc01703 in LUAD metastasis, particularly its role in regulating metastasis-related exosomes, have not been investigated. Our current study identified Linc01703 as a metastasis-inhibiting lncRNA in LUAD that promotes the secretion of CD81^+^ exosomes through the formation of the Rab27a/SYTL1/CD81 transport complexes. Mechanistically, Linc01703 enhanced the interaction between these proteins, leading to the increased secretion of CD81^+^ exosomes and modulation of immune cell infiltration. Additionally, we observed decreased CD81 in lung cancer tissues, which correlates with a poor prognosis in patients. Overall, our findings highlight the significance of Linc01703 in LUAD metastasis and its role in regulating CD81^+^ exosome secretion through the formation of the Rab27a/SYTL1/CD81 complex.

## 2. Materials and Methods

### 2.1. Clinical Specimens

This retrospective study was carried out using the case series of the First Affiliated Hospital of Sun Yat-sen University. All of the clinical tissue samples and histopathologically determined diagnoses were obtained from the First Affiliated Hospital of Sun Yat-sen University. The tumor–node–metastasis (TNM) classification from the Union for International Cancer Control (UICC) was used to evaluate the histological characteristics and clinicopathologic staging of the tumor samples. Nearby noncancerous lung samples were collected from neoplastic tissues that were excised from LUAD patients at a standard distance of 3 cm.

### 2.2. Animal Models

Female BALB/c-nu mice (5–6 weeks of age, 18–20 g) were housed in specific pathogen-free (SPF) facilities under a 12 h light/dark cycle at a temperature of 18–22 °C and humidity of 50–60%. To investigate the effects of Linc01703 and CD81 on LUAD metastasis, the indicated cells were inoculated via the tail vein or spleen. Metastasis was monitored via bioluminescence imaging (BLI). Briefly, mice were administered D-luciferin (150 mg/kg i.v., 10 min before imaging), anesthetized (2.5% isoflurane), and imaged with the Xenogen IVIS Spectrum Imaging System. Images were analyzed with Spectrum Living Image Software (Version 4.2). At the designated experimental endpoints, the mice were anesthetized and sacrificed, and the tumors were resected. All animal studies were approved by the SYSU Institutional Animal Care and Use Committee.

### 2.3. Cell Culture

LUAD cell lines, including the A549, NCI-H1975 (H1975), and noncancerous HEK293T (293T) cell lines, were obtained from the Cell Bank of Shanghai Institutes of Biological Sciences (Shanghai, China) or ATCC. The cells were cultured in Dulbecco’s modified Eagle’s medium (DMEM) supplemented with 10% fetal bovine serum (FBS) and 1% penicillin/streptomycin (penicillin 100 U/mL and streptomycin 10 μg/mL) at 37 °C in 5% CO_2_ in a humid atmosphere.

### 2.4. Plasmids, Virus Production, and Transfection

For the depletion of CD81, two single guide RNA sequences were cloned and inserted into PX458M pSpCas9-2A-GFP-MCS. For the depletion of Rab27a and SYTL1, two human shRNA sequences were cloned and inserted into pSuper-retro-puro retroviral vectors (Addgene, Watertown, MA, USA). Sequences of sgRNAs and shRNAs are listed in Supplementary Table S1. The full length of Linc01703 was cloned and inserted into pcDNA3-puro lentiviral vectors (Addgene), and the open reading frames (ORFs) of Rab27a, Rab27b, SYTL1, SYTL2, SYTL3, and CD81 was cloned and inserted into pSin-EF2-puro lentiviral vectors (Addgene). Stable cell lines for the overexpression of Linc01703 and the silencing of Rab27a and SYTL1 in A549 and H1975 cells were generated via lentiviral or retroviral infection using 293T cells and selected with puromycine (Sigma, St. Louis, MO, USA) antibiotics. After 2 weeks, the total mRNA and protein of the indicated tumor cells were collected and validated using real-time PCR and Western blotting assays. The transfection of the plasmids used in function assays and protein and RNA analysis was performed using a Lipofectamine 3000 reagent (Invitrogen, Waltham, MA, USA).

### 2.5. RNA Extraction and Real-Time PCR

Total RNA was isolated from cultured cells using a TRIzol reagent according to the instructions. cDNA was synthesized from 2 μg of total RNA with random primers using gene expression assays (Vazyme, Nanjing, China) and analyzed using Bio-Rad CFX Manager software (Version 3.0). The expression of Linc01703 and the indicated target mRNAs was assessed based on the threshold cycle (CT), and after normalization to GAPDH or β-actin expression, relative expression levels were calculated as follows: 2^−[(Ct of mRNA) − (CT of β-actin)]^. The sense and antisense primers that were used for quantitative reverse transcriptase PCR are listed in Supplementary Table S2. The experiments were performed at least three times, with triplicate replicates.

### 2.6. 5′ and 3′ Rapid Amplification of cDNA Ends (RACE)

Total RNA was extracted from human A549 cells with the TRIzol reagent. 5′ and 3′ RACE assays were carried out to determine the transcriptional initiation and termination sites of Linc01703 using a RACE Kit. The 5′ and 3′ RACE primers for Linc01703 are listed below:

5′RACE-1: 5′-CTTCACCCACTCGGCAGGAT-3′;

5′RACE-2: 5′-GCTCACCCACTTCGCACCGT-3′;

3′RACE-1: 5′-ATCCTGCCGAGTGGGTGAAG-3′;

3′RACE-2: 5′-CCTGCCGAGTGGGTGAAGCG-3′.

### 2.7. Cell Nucleus/Cytoplasm Fraction Isolation

Cell nucleus/cytoplasm fraction isolation was performed using a Nuclear and Cytoplasmic Extraction Kit (AM1921, Thermo Fisher, Waltham, MA USA) according to the supplier’s recommendation.

### 2.8. Western Blotting (WB) Analysis

In this study, WB analysis was performed according to a standard method. The primary antibodies that were used for WB analysis included anti-CD9 (Proteintech, Wuhan, China, 60232-1-Ig, 1:2000), anti-CD63 (Proteintech, 67605-1-Ig, 1:2000), anti-CD81 (Proteintech, 66866-1-Ig, 1:2000), anti-P84 (Proteintech, 10920-1-AP, 1:2000), anti-HA (Proteintech, 51064-2-AP, 1:5000), and anti-Flag (Proteintech, 20543-1-AP, 1:5000) antibodies. Blotted membranes were stripped and reblotted with anti-β-Actin (Proteintech, 20536-1-AP, 1:5000) antibodies used as loading controls. For exosomes from cancer cells, Ponceau S staining was used as a loading control.

### 2.9. TRSA RNA Pull-Down Assay

In order to explore the interaction between Linc01703 and Rab27a, Rab27b, SYTL1, SYTL, and CD81, we performed tRSA RNA pull-down assays (Thermo Fisher, 20164) as previously described [28,29]. Briefly, the Linc01703 and Linc01703 antisense were reverse-transcribed via PCR with T4 RNA polymerase using the RNA 3′ End Desthiobiotinylation Kit (Thermo Fisher, 20163), and then the labeled RNAs were purified using the RNA Purification Kit (Thermo Fisher, K0731). Then, 50 pmol of biotein-labeled RNAs were mixed with the cell lysis of 293T cells and incubated for 3–4 h at 4 °C. After incubation, streptavidin magnetic beads were added to each binding reaction and further incubated at 4 °C overnight. Then, the proteins that did not bind with beads were washed with wash buffer five times. Finally, the beads were boiled in a loading buffer, and the binding proteins of Linc01703 were resolved via a Western blotting assay.

### 2.10. Wound Healing, Invasion Assays, and MTT Assay

Tumor cells were plated in 6-well plates at 1 × 10^6^ cells per well for wound healing assays. After 24 h of the indicated treatment, the cells were scratched with a pipette tip. At the designated times, cell migration was observed and captured on a camera. For cell invasion assays, Transwell chambers were used. The indicated cells were subsequently trypsinized and resuspended in FBS-free DMEM. The lower chambers of the Transwell plates were then filled with 500 μL of DMEM supplemented with 10% FBS as a chemoattractant, and the cells were plated in the upper chambers. Cells that had crossed to the bottom side of the inserts were fixed, stained with 0.1% Crystal violet (Sangon, Shanghai, China, A600331), photographed, and quantified by counting the cells in 5 random fields after a 24 h incubation period. A total of 2000 tumor cells were seeded in 96-well plates for the MTT experiment. Then, 20 μL of the MTT solution (Sigma) was added to each well after 24 h of the indicated treatment, and the wells were then incubated for 4 h at 37 °C in 5% CO_2_ in a humid environment. After the MTT reagent was discarded, 150 μL of dimethyl sulfoxide (DMSO, Sigma) was added to each well to completely dissolve the crystals. The absorbance values of each well were measured at OD_490nm_.

### 2.11. Exosome Isolation and Nanoparticle Tracking Analysis (NTA)

For exosome isolation, the indicated cells were plated in 100 mm Petri dishes at 1 × 10^6^ cells and cultured for 24 h in DMEM and for 48 h in exosome-depleted DMEM. Two days later, the supernatant from each dish was collected and processed via centrifugation, ultrafiltration, and ultracentrifugation. Briefly, to remove cell fragments, the medium was centrifuged at 300× *g* for 10 min, 2000× *g* for 10 min, and 12,000 rpm for 20 min. Subsequently, the centrifuged supernatant was concentrated using an ultrafiltration tube (Millipore, Burlington, MA, USA, UFC9100). After ultrafiltration, the concentrate was resuspended in 9 mL of phosphate-buffered saline (PBS) in an ultracentrifuge tube (Beckman, Brea, CA, USA, 326823) and centrifuged twice at 120,000× *g* for 2 h and 70 min. Finally, the exosome pellets were resuspended in 200 μL of PBS for the following experiments. The concentration of the exosomes in A549-Vector and A549-Linc01703 were analyzed using a NanoSight NS300 (NanoSight Technology, Malvern, UK) equipped with a 488 nm laser at a camera level of 10 and a detection threshold of 7. The expression of the exosome markers CD81, CD9, and CD63 was measured via Western blotting after the protein concentration of the exosomes was measured using the BCA protein assay kit (Thermo Fisher, 23225).

### 2.12. Flow Cytometry Analysis

Subcutaneous tumor tissues were dissociated into single cells using a mortar to evaluate the effect of CD81^+^ exosomes and Linc01703 on immune cells infiltrating the TME. PBS was used to wash and suspend the cells from the tumor tissue before they were stained for 30 min with anti-CD11b, anti-Ly6G, anti-B220, anti-CD86, anti-CD3, anti-CD49, and anti-CD80 antibodies. The CytExpert software (Version 2.3)was used to collect data on Cytoflex (Beckman Coulter) and evaluate it.

### 2.13. RNA Sequencing

Total RNA samples from A549-Vector and A549-Linc01703 cells were extracted for RNA sequencing, which was carried out commercially by the Berry Genomics Corporation following standard Agilent protocols. The RNA sequencing data are in Supplementary Table S3, which includes the mRNA expression profiling of A549-Vector and A549-Linc01703 cells and the 199 up-regulated genes in A549-Linc01703 cells.

### 2.14. Statistical Analysis

Except for the sequencing data, all statistical analyses were performed using GraphPad Prism 8, version 8.3.0 (GraphPad Software, San Diego, CA, USA). Comparisons between two groups were performed using a two-tailed *t*-test, whereas two-way ANOVA tests were used for comparing multiple treatments with a control group. All error bars show the mean  ±  SD derived from three independent experiments. In each case, *p* <  0.05 was considered statistically significant.

## 3. Results

### 3.1. Linc01703 Expression Is Decreased in Metastatic Lung Cancer Cells

**Figure 1:**
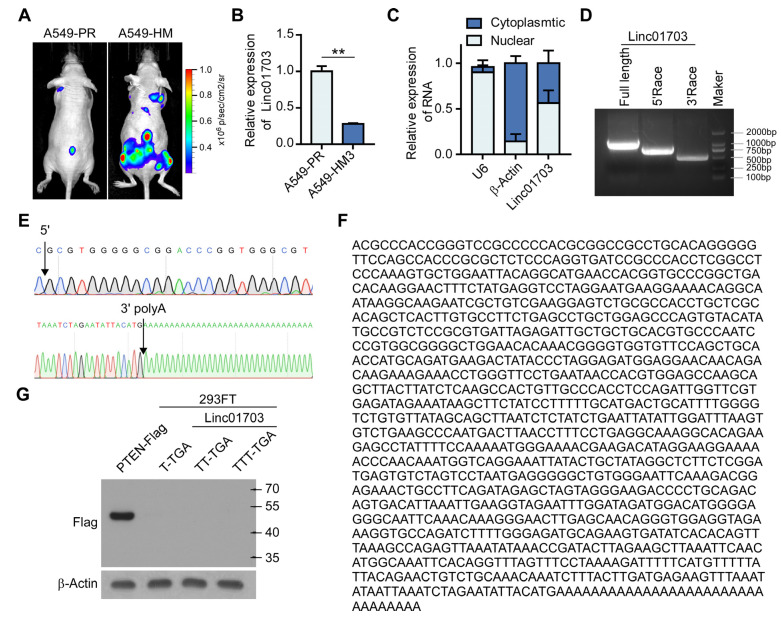
Linc01703 expression is decreased in metastatic lung cancer cells. (**A**) Indicated A549 cells (1 × 10^6^) were injected via ventricle, and representative bioluminescent images of metastasis are shown. (**B**) Relative fold change of Linc01703 in A549-PR and A549-HM cells. (**C**) The relative expression of U6 (nuclear control) and β-Actin (cytoplasmic control) and the expression of Linc01703 were analyzed by using qRT-PCR in the nuclear and cytoplasmic fractions. (**D**) The images of PCR products from the 5′-RACE and 3′-RACE procedures. (**E**,**F**) The sequence of the full length of Linc01703. Blue: C, Black: G, Red: T, Green: A. (**G**) The full length of Linc01703 was cloned into pLenti with an ATG-start codon and a C-terminal Flag peptide in three expression patterns. An anti-Flag antibody was used to probe transcribed proteins. The uncropped blots are shown in File S1. PTEN with a Flag tag served as a positive control. Results are presented as mean ± SD, ** *p* < 0.01.

To investigate the process of LUAD metastasis, we utilized an in vivo three-stage selection strategy [30] to establish a LUAD metastasis model and derived a highly metastatic subpopulation from A549 cells, referred to as A549-highly metastatic cells (A549-HM3). Compared with the parental (A549-PR) cells, A549-HM3 exhibited significantly enhanced metastatic potential, as evidenced by bioluminescence imaging (BLI) analysis in mice (Figure 1A). Impressively, we observed a significant reduction in the expression of LincRNA01703 (ENST00000656201.1, Linc01703) in A549-HM3 cells compared to A549-PR cells (Figure 1B). Cellular fractionation assays revealed the presence of Linc01703 in both the cytoplasm and nucleus of A549 cells (Figure 1C). Furthermore, the rapid amplification of cDNA ends (RACE) experiment confirmed the full-length sequence of Linc01703, which consisted of 1159 nucleotides and a 3′ polyA tail (Figure 1D–F). Importantly, an analysis of the coding potential strongly suggested that Linc01703 lacks protein-coding capacity (Figure 1G). Collectively, these data provide compelling evidence that Linc01703 is significantly downregulated in metastatic LUAD, prompting further investigation into its potential role in LUAD metastasis.

### 3.2. Linc01703 Inhibits Lung Cancer Metastases In Vivo

**Figure 2:**
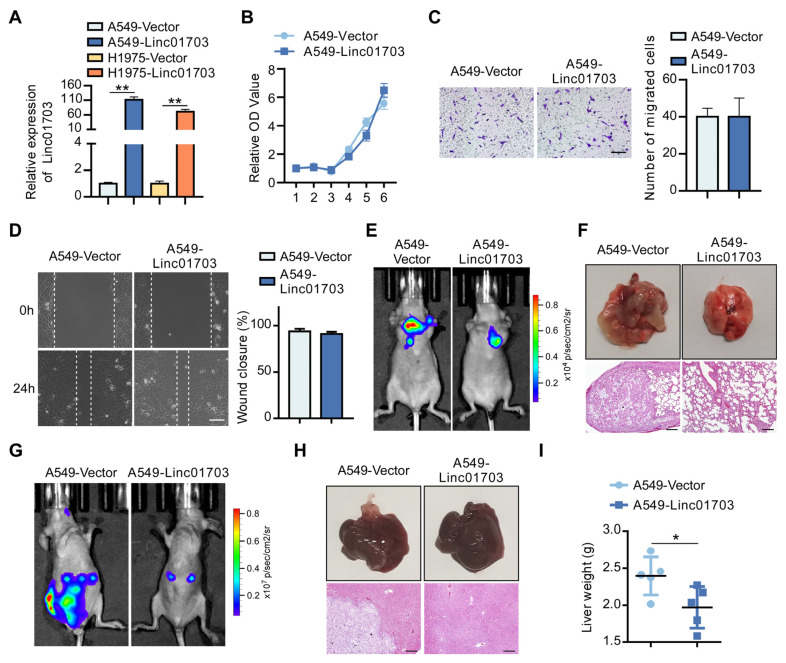
Linc01703 inhibits lung cancer metastases in vivo. (**A**) Relative fold change of Linc01703 in Vector and Linc01703-overexpressing cells of A549 and H1975. (**B**) MTT assay of A549-Vector and A549-Linc01703 cells. (**C**) Representative graphs and quantification of indicated cells analyzed via Transwell assays. Scale bar: 100 μm. (**D**) Representative graphs and the statistical results of the wound healing assay in A549-Vector and A549-Linc01703 cells. Wound closures were photographed at 0 and 24 h after wounding. Scale bar: 100 μm. The percentage of wound closure was based on statistics in Image J. (**E**) Indicated A549 cells (1 × 10^6^) were injected via tail vein (**E**,**F**) or spleen (**G**,**I**), and representative bioluminescent images (BLI) (**E**,**G**), tumor picture (**F** upper panel; **H** upper panel), H&E staining (**F** lower panel; **H** lower panel, Scale bar: 100 μm), and liver weight (**I**) of metastasis are shown. (*n* = 5 per group). Results are presented as mean ± SD derived from three independent experiments, * *p* < 0.05, ** *p* < 0.01.

To investigate the role of Linc01703 expression in LUAD metastasis, we established stable Linc01703-overexpressing cell lines in A549 and H1975 (Figure 2A). The overexpression of Linc01703 in A549 and H1975 cells did not significantly affect the proliferative and invasive capabilities of LUAD cells in vitro, as shown by MTT, Transwell, and wound healing assays (Figure 2B–D). However, in the mouse metastasis model, injection of Linc01703-overexpressing A549 cells via the lateral tail vein or spleen resulted in significantly reduced colonization and metastasis in the lung (Figure 2E,F) and liver (Figure 2G–I) of mice compared to A549-Vector cells. Taken together, these data demonstrate that Linc01703 acts as an inhibitor of LUAD cell metastasis in vivo.

### 3.3. Linc01703 Promotes the Release of CD81^+^ Exosomes

**Figure 3:**
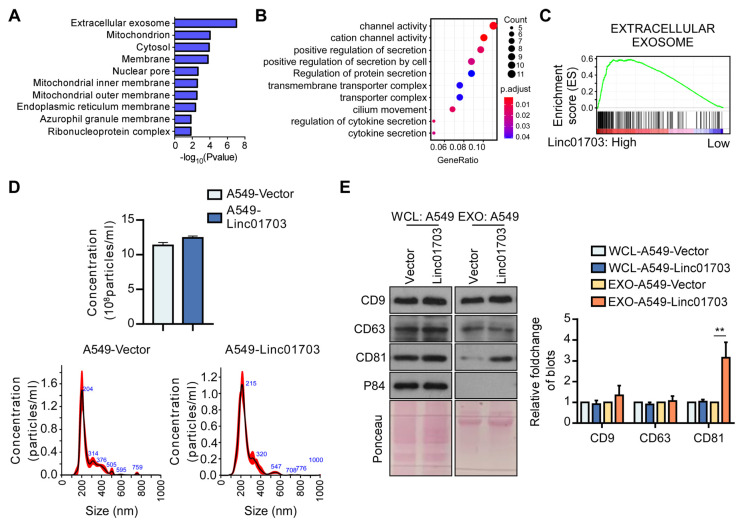
Linc01703 promotes the release of CD81^+^ exosomes. (**A**,**B**) Gene Ontology (GO) and Kyoto Encyclopedia of Genes and Genomes (KEGG) analyses of the 199 upregulated genes in A549-Linc01703 compared with A549-Vector genes. (**C**) Gene set enrichment analysis (GSEA) of the correlation between Linc0170 and extracellular exosome genes using the TCGA lung cancer database. (**D**) Concentration of exosomes in A549-Vector and A549-Linc01703 cells. (**E**) The effect of Linc01703 overexpression on CD9, CD63, and CD81 in whole cell lysis (WCL) and exosome (EXO). The uncropped blots are shown in File S1. ** *p* < 0.01.

To further understand the mechanism by which Linc01703 inhibits metastasis, we performed RNA sequencing and Gene Ontology (GO) and Kyoto Encyclopedia of Genes and Genomes (KEGG) analyses on A549-Linc01703 and A549-Vector cells. The analysis revealed that the most enriched annotations among the 199 upregulated genes in A549-Linc01703 cells were related to extracellular exosomes (Figure 3A,B). We also observed a positive correlation between the expression of Linc01703 and extracellular exosome genes in the Cancer Genome Atlas (TCGA) lung cancer datasets (Figure 3C). Furthermore, we isolated and analyzed exosomes from A549-Linc01703 and A549-Vector cells and found unchanged extracellular nanoparticle counts (Figure 3D). However, the expression of CD81, a major constituent of the exosome, increased, while the expression of other exosome markers, like CD9 and CD63, remained unchanged (Figure 3E). Considering the heterogeneity of exosome subtypes and marker expression, this increase in CD81 could potentially reflect an increase in the number of CD81^+^ exosomes and suggest that Linc01703 promotes the release of CD81^+^ exosomes instead of increasing the concentration of exosomes.

### 3.4. Linc01703 Inhibits Lung Cancer Metastasis and Affects Immune Cell Infiltration through CD81^+^ Exosomes

**Figure 4:**
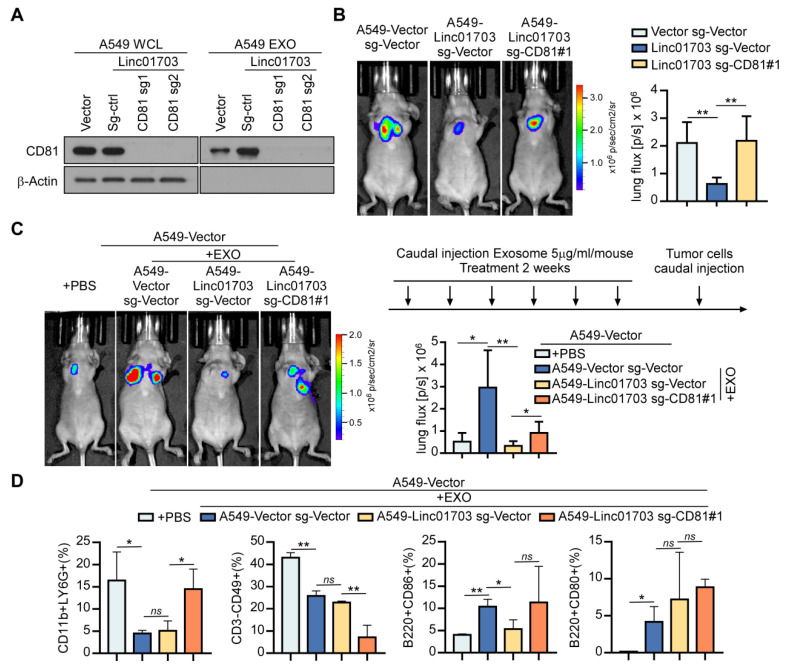
Linc01703 inhibits lung cancer metastasis and affects immune cell infiltration through CD81^+^ exosomes. (**A**) The expression of CD81 in the indicated cells. The uncropped blots are shown in File S1. (**B**,**C**) Indicated A549 cells (1 × 10^6^) were injected via tail vein, and representative bioluminescent images of metastasis are shown (n = 5 per group). (**D**) Percentage of CD11b^+^Gr-1^+^, B220^+^CD86^+^, CD3^−^CD49^+^, and B220^+^CD80^+^ cells in the tumor microenvironment after exosome treatment. Results are presented as mean ± SD derived from three independent experiments, * *p* < 0.05, ** *p* < 0.01, ns: no significance.

To investigate the functional significance of the CD81^+^ exosome in the Linc01703-mediated inhibition of metastasis, we silenced CD81 in A549-Vector and A549-Linc01703 cells (Figure 4A). Notably, silencing CD81 reversed the metastasis-suppressive effect of Linc01703 in a tail vein injection mouse model (Figure 4B). Moreover, we next studied the roles of exosomes derived from the indicated A549 cells in lung cancer cell metastases. Mice were caudally injected with exosomes 2 weeks before a caudal injection of A549-Vector cells, and cellular metastasis was evaluated with a mouse BLI assay (Figure 4C). Moreover, we found that exosomes derived from A549-Linc01703 cells reduced the metastasis of lung cancer cells compared to the exosomes from A549-vector cells. However, when CD81 was silenced, the inhibitory effect of Linc01703 on metastasis was abolished (Figure 4C). Additionally, we observed that the exosomes from A549-Vector cells altered immune cell infiltration in the tumor microenvironment by reducing the proportions of neutrophils (CD11b^+^Gr-1^+^) and NK cells (CD3^-^CD49^+^) and increasing the proportion of CD86^+^ B cells (B220^+^CD86^+^) and CD80^+^ B cells (B220^+^CD80^+^) compared with PBS treatment (Figure 4D). In particular, the administration of exosomes from A549-Linc01703 cells restained the infiltration of CD86^+^ B cells, while silencing CD81 reversed these effects induced by Linc01703 (Figure 4D). CD86^+^ B cells, a physiologic B cell subset, have been shown to be increased in the tumor microenvironment of gastric cancer [31,32], colorectal cancer [33], and head and neck squamous cell carcinoma [34] patients. Therefore, these data suggest that the inhibition effect of Linc01703 in LUAD metastasis may be dependent on the infiltration of CD86^+^ B cells via CD81^+^ exosomes.

### 3.5. Linc01703 Promotes the Formation of Rab27a/SYTL1/CD81 Transport Complexes

**Figure 5:**
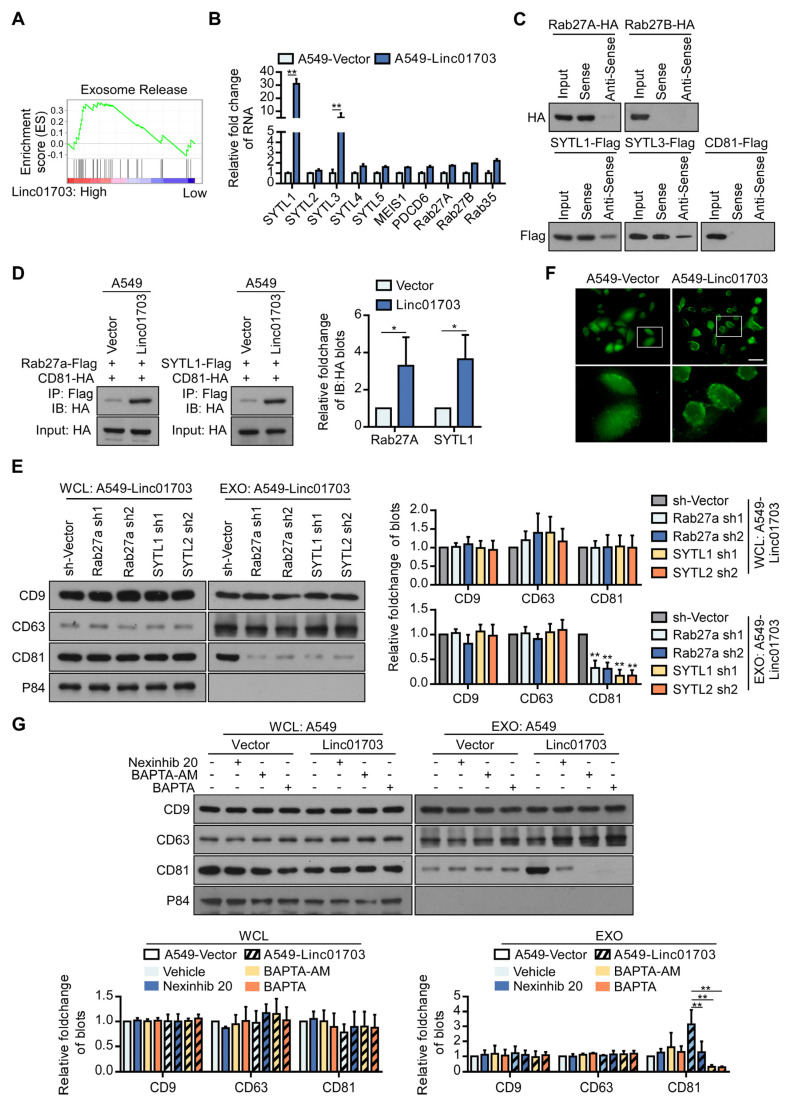
Linc01703 promotes the formation of Rab27a/SYTL1/CD81 transport complexes. (**A**) Gene set enrichment analysis (GSEA) of the correlation between Linc01703 and extracellular exosome genes using genes in A549-Linc01703 and A549-Vector cells. (**B**) Relative fold change of exosome release genes in A549-Linc01703 and A549-Vector cells. (**C**) RNA pull-down assay and Western blot assay validation of the binding of Rab27a, Rab27b, SYTL1, SYTL3, and CD81 with Linc01703. (**D**) The effect of Linc01703 on the binding of Rab27a with CD81 and CD81. (**E**) The effect of silencing Rab27a or SYTL1 on the expression of CD9, CD63, and CD81 in WCL and EXO. (**F**) Fluo-4 AM staining of indicated cells depicts intracellular calcium. Scare bar: 50 μm. (**G**) The effect of Nexinhib 20 (the inhibitor of Rab27a-SYTL1 binding), BAPTA-AM (Ca^2+^ chelator), or BAPTA (Ca^2+^ chelator) on the expression of CD9, CD63, and CD81 on the WCL and EXO of the indicated cells. The uncropped blots are shown in File S1. Results are presented as mean ± SD, * *p* < 0.05, ** *p* < 0.01.

Previous studies have extensively shown that lncRNAs perform their functions by physically interacting with their binding proteins. To explore the molecular mechanism by which Linc01703 promotes the release of CD81^+^ exosomes, we investigated the interaction proteins of Linc01703. Among the genes related to exosome release, we found that synaptotagmin-like 1 (SYTL1) was upregulated in A549-Linc01703 cells (Figure 5A,B). Interestingly, SYTL1 has been shown to specifically bind to Rab27a, a GTPase involved in exosome release [35,36]. We confirmed the interaction of Linc01703 with Rab27a, SYTL1, and CD81 using RNA pull-down and Western blotting assays. And we found that Linc01703 could bind with Rab27a and SYTL1 (Figure 5C). Furthermore, the overexpression of Linc0703 enhanced the interaction of Rab27a with CD81 and SYTL1 (Figure 5D). Moreover, the inhibition of Rab27a or SYTL1 reversed the increased release of CD81^+^ exosomes induced by Linc01703 in A549 cells (Figure 5E). Additionally, we observed an increase in intracellular calcium (Ca^2+^) levels near the membrane in A549-Linc01703 cells, which is known to be involved in the release of exosomes (Figure 5F). Thus, we investigated the treatment of Nexinhib 20, an inhibitor of Rab27a-SYTL1 protein—protein interactions, and intracellular calcium chelators (BAPTA-AM and BAPTA), which significantly reduced the expression of CD81 in the exosomes derived from A549-Linc01703 cells compared with the control-treated group (Figure 5G). Taken together, our data indicate that Linc01703 promotes the formation of Rab27a/SYTL1/CD81 transport complexes through the upregulation of SYTL1 in LUAD cells.

### 3.6. CD81 Is Decreased in Lung Cancer Tissues and Correlates with Better Prognosis of Patients

**Figure 6:**
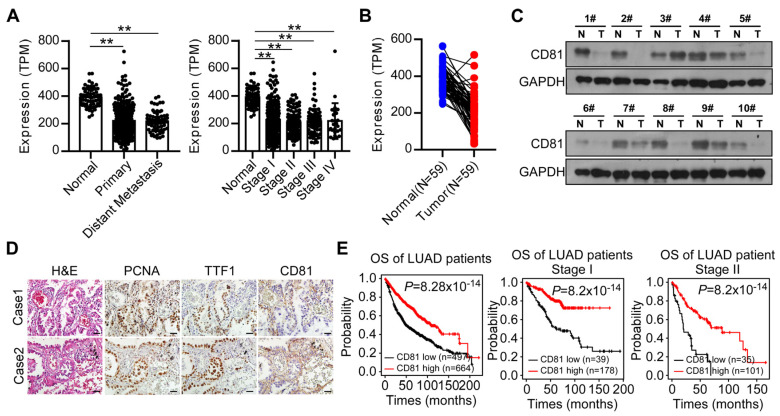
CD81 is decreased in lung cancer tissues and correlates with a better prognosis for patients. (**A**) Analyses of the expression levels of CD81 in patients and control using the TCGA LUAD dataset (two-tailed paired Student’s *t*-test). (**B**) Analyses of the expression levels of CD81 in paired LUAD and adjacent non-tumor tissues (n = 59) using the TCGA dataset (two-tailed paired Student’s *t*-test). (**C**) A Western blot assay shows the expression of CD81 in paired LUAD and adjacent non-tumor tissues (n = 10). The uncropped blots are shown in File S1. (**D**) Representative images of IHC from LUAD patients show the protein expression level of CD81. Scale bar: 20 μm. (**E**) Kaplan–Meier analyses of overall survival (OS) based on the KMPLOT LUAD dataset on CD81 expression. Results are presented as mean ± SD, ** *p* < 0.01.

To further investigate whether the above findings were clinically relevant, we examined the expression of CD81 in clinical LUAD tissue samples and determined its correlation with patient prognosis. As shown in Figure 6A,B, we found that CD81 expression was significantly decreased in LUAD tumor tissues, especially in tumor tissues with local or distant metastasis. CD81 protein levels were also downregulated in eight LUAD tissue samples in the Western blotting assay, as shown by weak immunostaining in LUAD tissue specimens compared with adjacent benign lung tissue specimens through immunohistochemical (IHC) assay (Figure 6C,D). Additionally, low-level CD81 expression was associated with shorter overall survival (OS), especially in stage I and II patients (Figure 6E). The cutoff value for distinguishing high versus low expression was selected using receiver operating characteristic (ROC) curve analysis. Together, these clinical data reveal that CD81 is significantly downregulated in LUAD tissues and may play an essential role during LUAD metastasis.

## 4. Discussion

Recent research has highlighted the involvement of lncRNAs in various tumorigenic processes, including tumor cell proliferation, invasion, metabolism, and immunological function in the tumor microenvironment (TME) [37,38]. However, the link between lncRNAs and the regulation of exosome secretion from tumor cells remains poorly understood. In the current study, we investigated the role of Linc01703, a downregulated lncRNA in metastatic lung cancer cells, in promoting exosome secretion and inhibiting LUAD metastasis. 

We first performed GO, KEGG, and GSEA analyses to analyze the RNA sequencing data from A549-Linc01703 cells. These analyses revealed an enrichment of genes related to exosome secretion in A549-Linc01703 cells. Furthermore, nanoparticle tracking analysis (NTA) and Western blotting analyses confirmed that the overexpression of Linc01703 promoted the release of CD81^+^ exosomes by LUAD cells, indicating a positive correlation between Linc01703 and CD81^+^ exosome secretion. Moreover, our study suggests a correlation between LUAD metastasis and the downregulation of Linc01703, which may be influenced by TGF-β and hypoxia. The functional mechanisms of lncRNAs are often determined by their subcellular location. It has been demonstrated that lncRNAs can regulate tumorigenic processes by interacting with RNA-binding proteins (RBPs). As Linc01703 was localized in both the cytoplasm and nucleus of A549 cells, we hypothesize that cytoplasmic Linc01703 may interact with Rab27a and SYTL1 to enhance the secretion of CD81^+^ exosomes, while the role of nuclear Linc01703 remains unknown and requires further investigation. 

Exosome secretion is a complex process involving the transport of multivesicular bodies (MVBs), docking, and fusion with the plasma membrane and is regulated by various molecules and pathways [9,39]. We investigated the mechanism underlying the secretion of CD81^+^ exosomes in LUAD cells, focusing on the role of Linc01703. The Rab family, including Rab5 [40], Rab7 [41], Rab11 [42], Rab27a [43,44], Rab27b [43,44], and Rab35 [45], has been demonstrated in previous studies to have essential roles in controlling MVB transport and affecting exosome release. Our results demonstrated that Linc01703 upregulated the expression of SYTL1, an effector of Rab27a [46]. Moreover, the overexpression of Linc01703 induced the interaction between Rab27a, SYTL1, and CD81, while silencing Rab27a or SYTL1 decreased the release of CD81^+^ exosomes induced by Linc01703. These findings suggest that Linc01703 regulates the release of CD81^+^ exosomes by modulating SYTL1 expression and the formation of Rab27a/SYTL1/CD81 complexes.

Indeed, extracellular vesicles, including microvesicles and exosomes, have been shown to play unique roles and mechanisms in intercellular communication and the tumor microenvironment [11,47]. In breast cancer, CD81^+^ exosomes released by fibroblasts have been implicated in promoting the migration of breast cancer cells through the interaction of CD81 and Wnt11 [23]. Similarly, in our study, we found that CD81, which is abundant in the exosome derived from A549-Linc01703 cells, can be secreted and play a biological role in the tumor microenvironment. Previous studies have shown that CD81 is involved in the activation of immune cells, including B cells [16,48,49], T cells [50,51], and NK cells [52], leading to an enhanced antitumor immune response. Interestingly, we observed that the exosomes derived from A549-Linc01703 decreased the infiltration of CD86^+^ B cells in the tumor microenvironment, while CD81 silencing reversed it. Additionally, the reduced expression level of CD81 in LUAD patients with metastasis and its correlation with a better prognosis further highlight the clinical relevance of the CD81^+^ exosome. Considering the important role of Linc01703-induced CD81^+^ exosome release, our findings provide new insights into the potential clinical application of CD81^+^ exosome-related cancer therapy.

In summary, our study provides evidence that Linc01703 promotes the formation of the Rab27a/SYTL1/CD81 complex, leading to the increased secretion of CD81^+^ exosomes. These exosomes subsequently reduced the proportion of infiltrating immune cells in the lung tumor microenvironment and impaired LUAD metastasis.

## Data Availability

The data associated with this paper are available upon reasonable request to the corresponding author. Reagents, antibodies, and resources are listed in the Supplementary Table.

## Supplementary Materials

The following supporting information can be downloaded at: https://www.mdpi.com/article/10.3390/cancers15245781/s1, File S1: Full pictures of the Western blots; Table S1: Oligos used for knockdown or knockout genes; Table S2: Sense and antisense primers used for qRT-PCR; Table S3: Original data for RNA seqencing.
