# Supplementary material for: LincRNA01703 Facilitates CD81+ Exosome Secretion to Inhibit Lung Adenocarcinoma Metastasis via the Rab27a/SYTL1/CD81 Complex
Source: Cancers (Basel). 2023 Dec 9;15(24):5781. doi: 10.3390/cancers15245781 (PMC10742068; doi:10.3390/cancers15245781)
Supplement: Supplementary file 1 [file cancers-15-05781-s001.zip › File S1 Full pictures of the Western blots.pdf]

Figure S1

G

Repeat 1

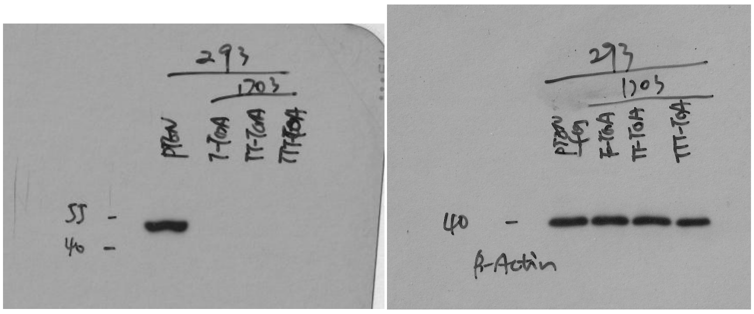

Repeat 2

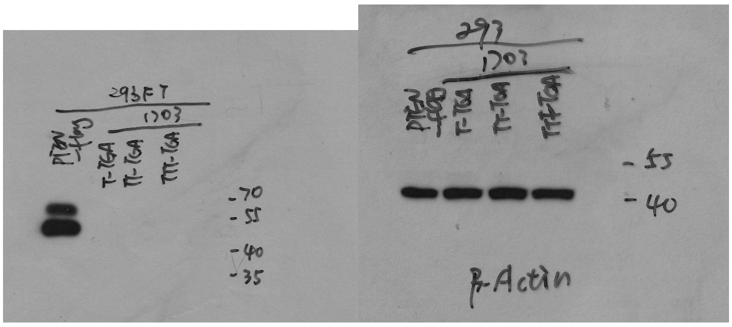

Repeat3

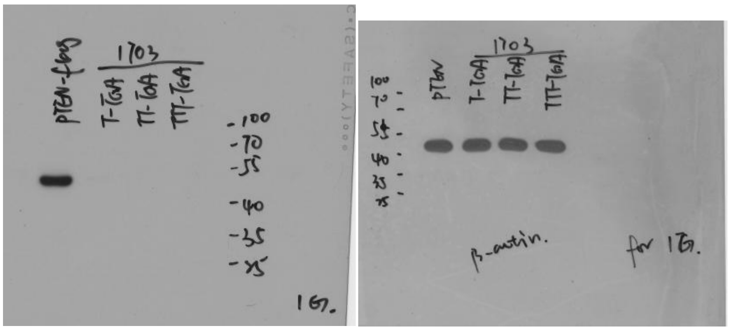

Figure S3

E

Repeat 1

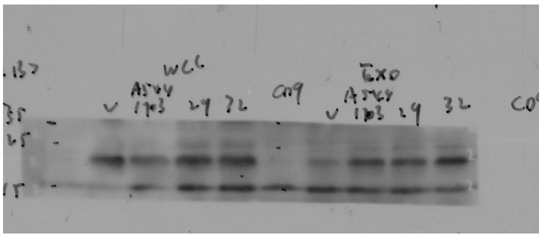

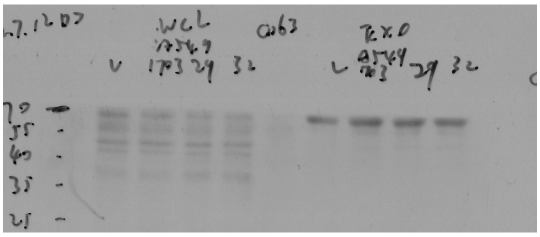

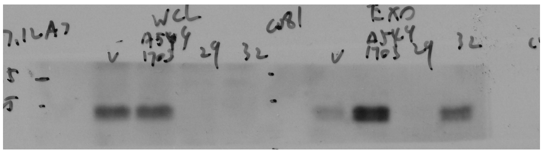

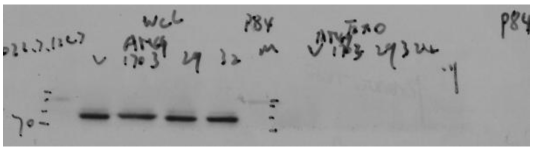

Repeat 2

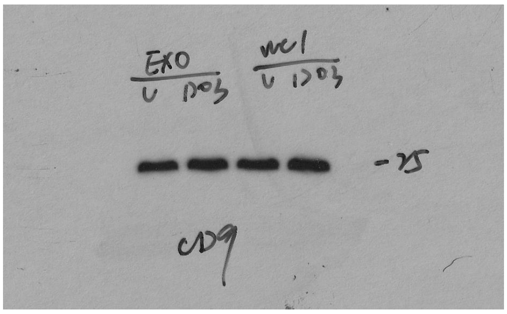

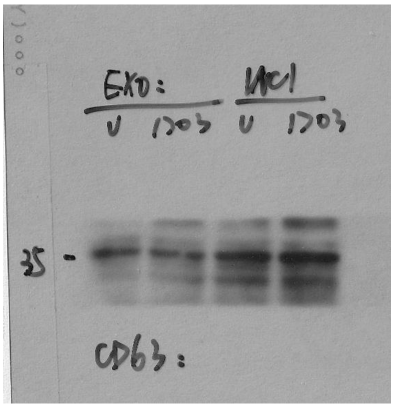

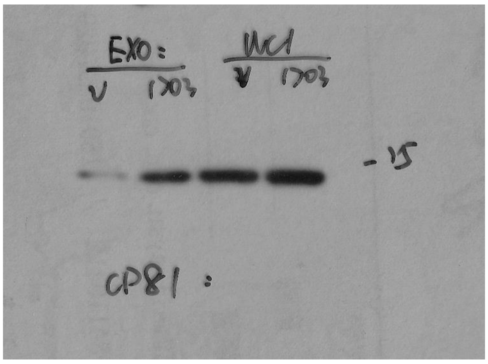

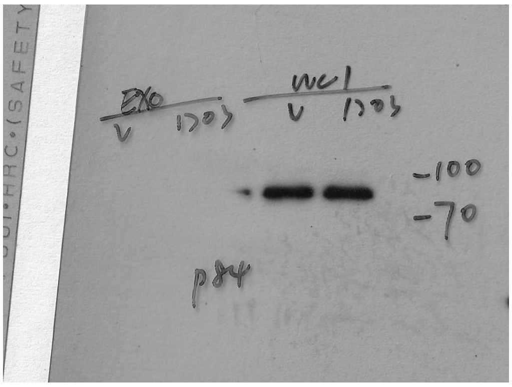

Repeat 3

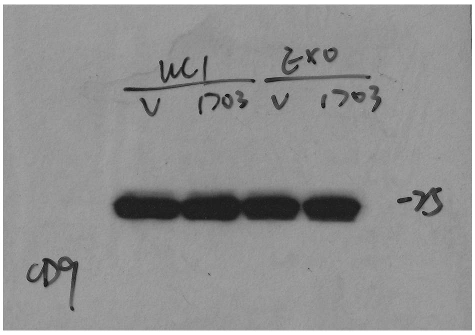

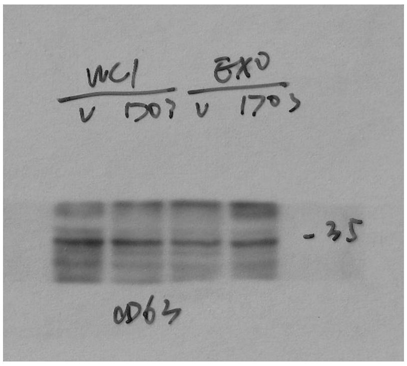

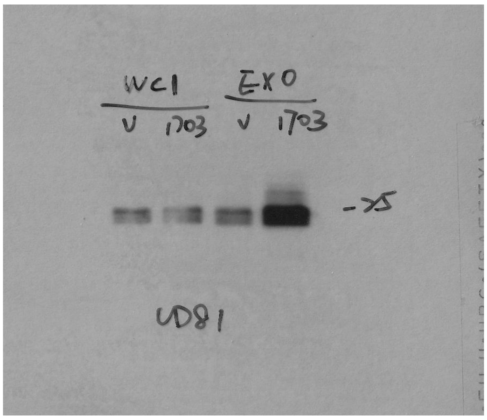

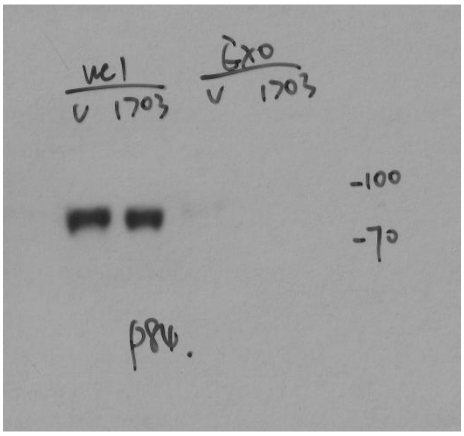

Figure S4

A

Repeat 1

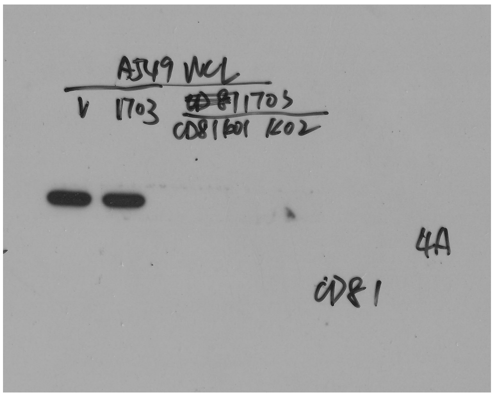

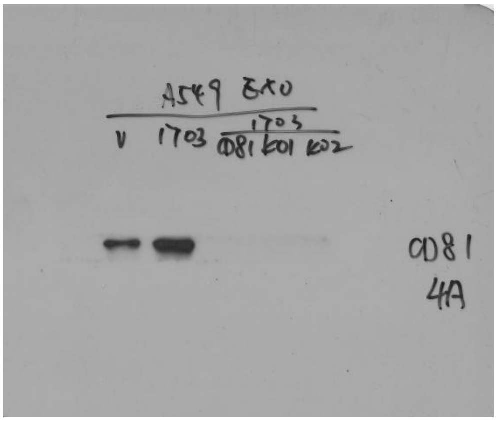

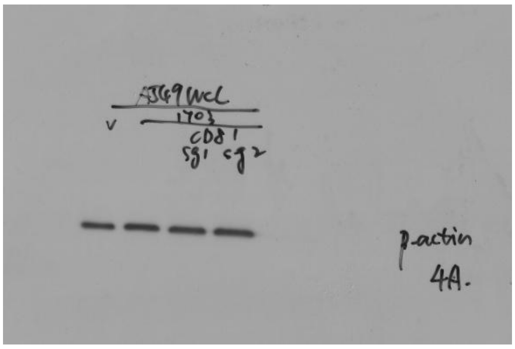

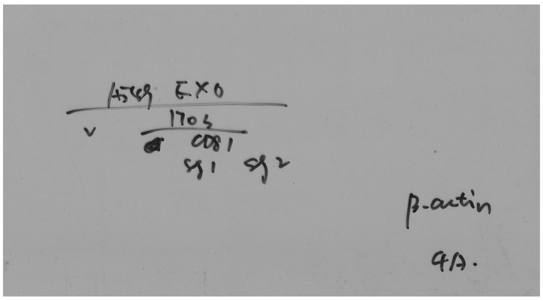

Repeat 2

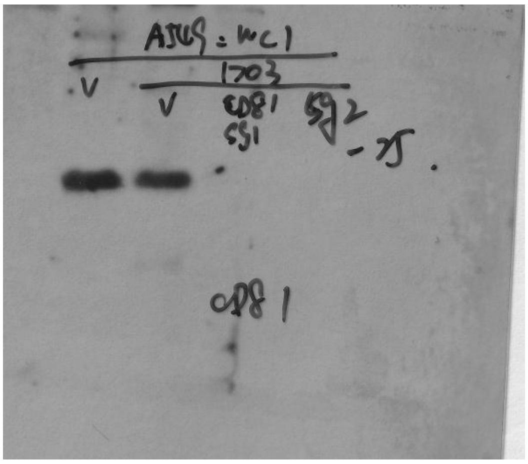

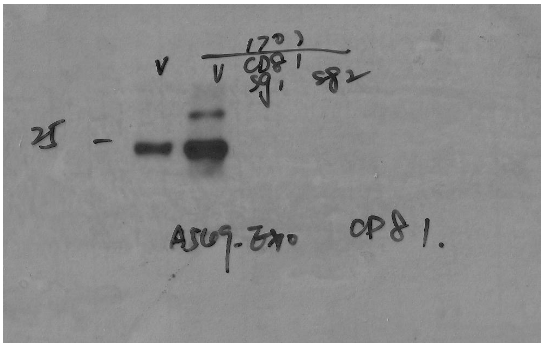

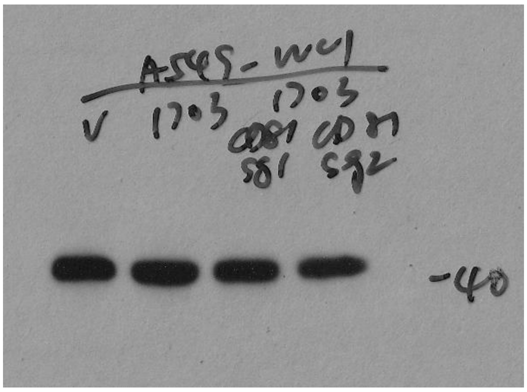

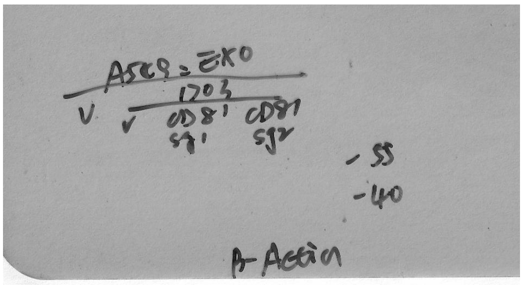

Repeat 3

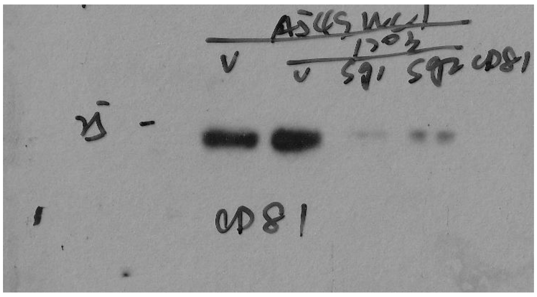

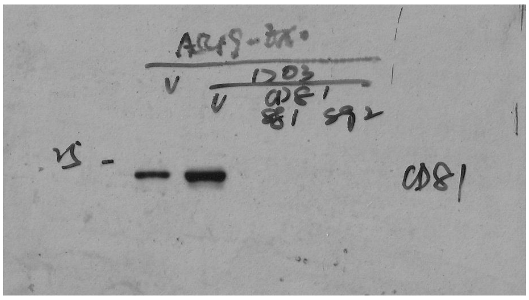

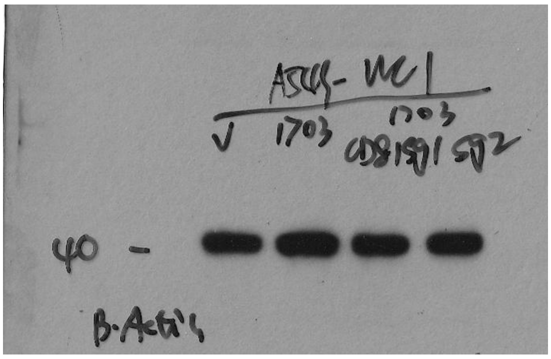

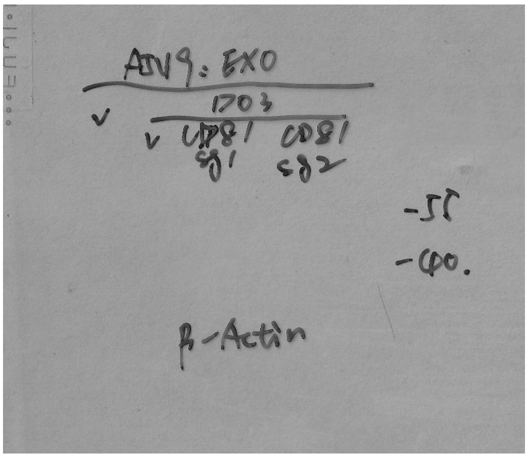

Figure S5

C

Repeat 1

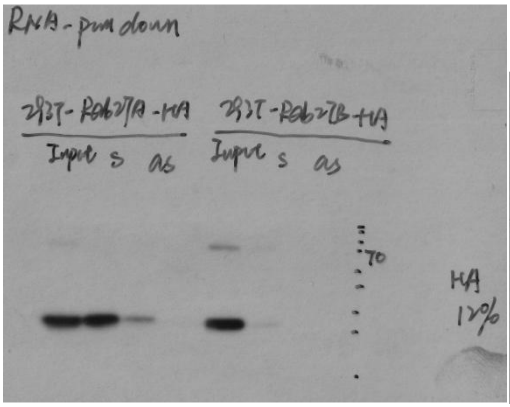

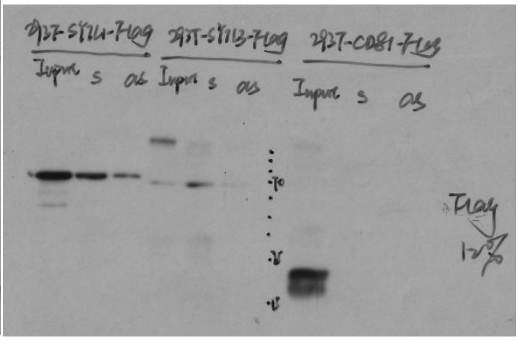

Repeat 2

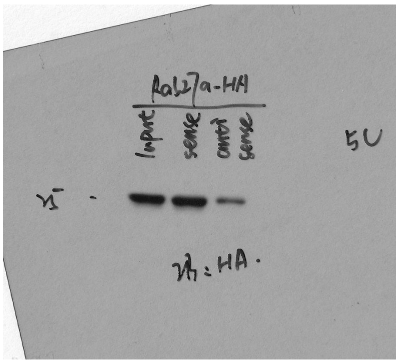

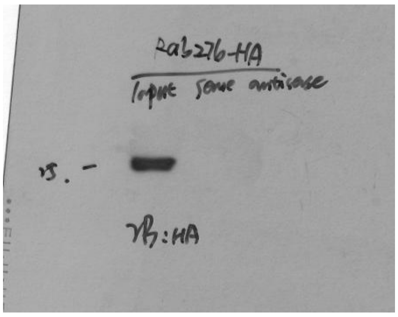

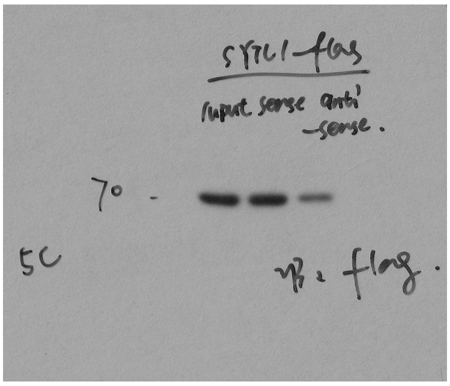
SYTL1-flag
Input sense anti-sense.
70 -
5C
IB: flag.

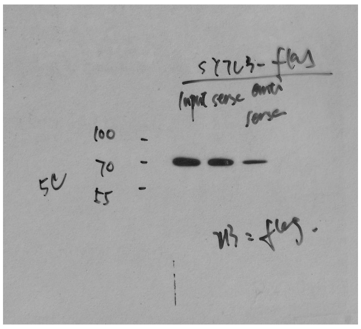
SYTL3-flag
Input sense anti sense
100 -
70 -
55 -
5C
IB: flag.

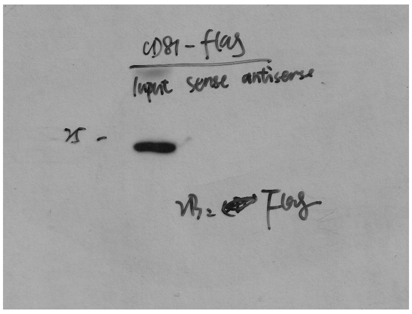
CD81-flag
Input sense antisense.
25 -
IB: Flag

Repeat 3

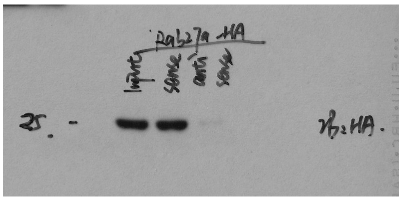
Rab27a-HA
Input sense anti sense
25 -
IB: HA.

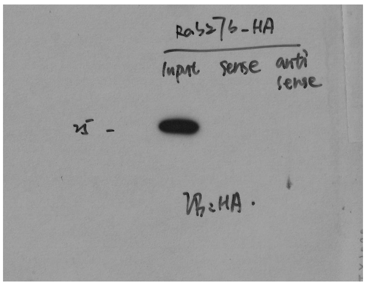
Rab27b-HA
Input sense anti sense
25 -
IB: HA.

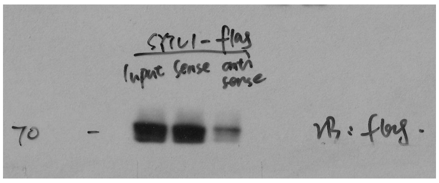
SYTL1-flag
Input sense anti sense
70 -
IB: flag.

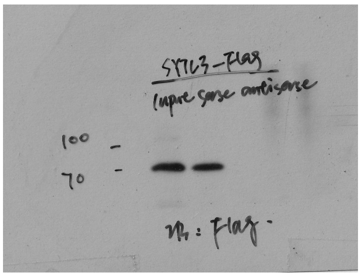
SYTL3-Flag
Input sense antisense
100 -
70 -
IB: Flag.

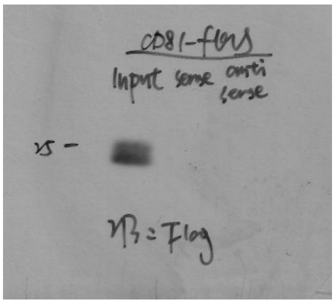
CD81-flag
Input sense anti sense
25 -
IB: Flag

Figure S5

D

Repeat 1

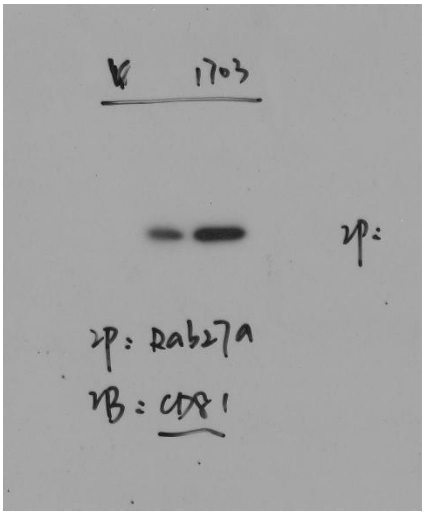

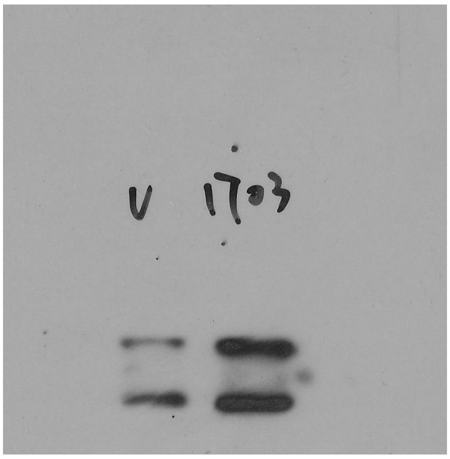

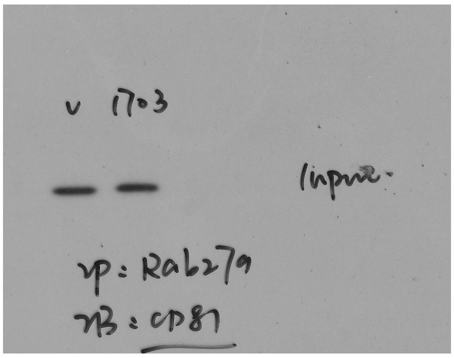

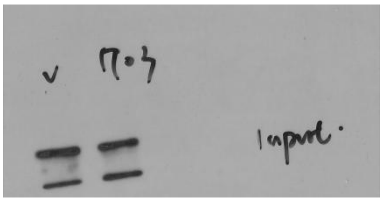

Repeat 2

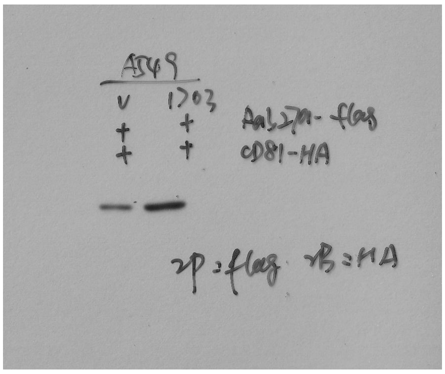

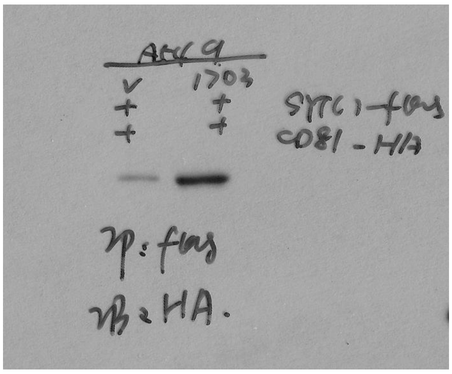

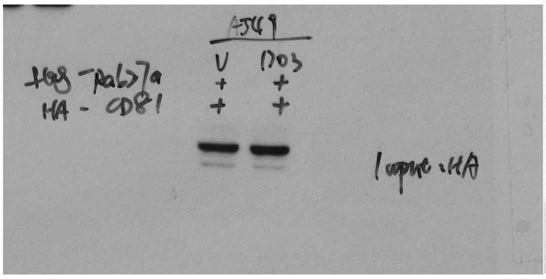

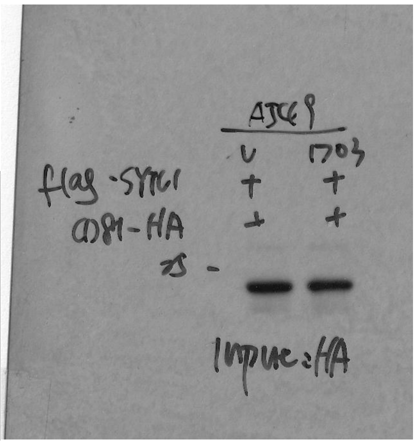

Repeat 3

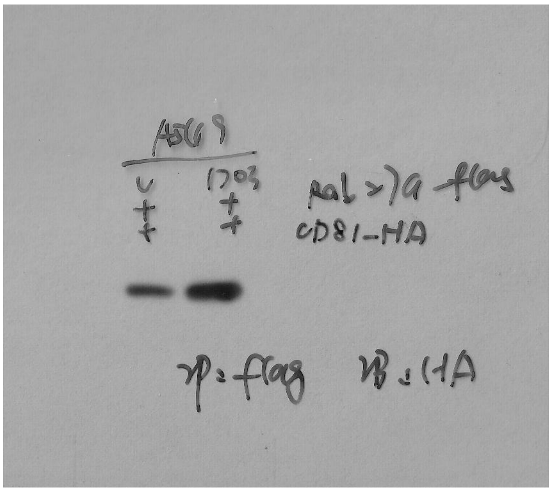

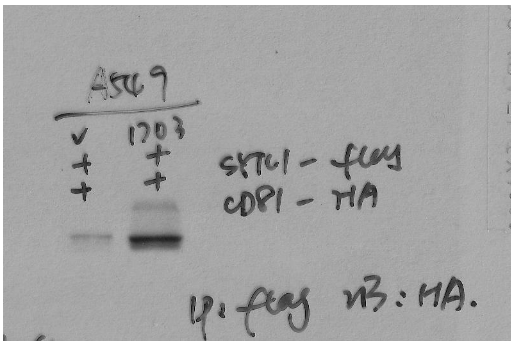

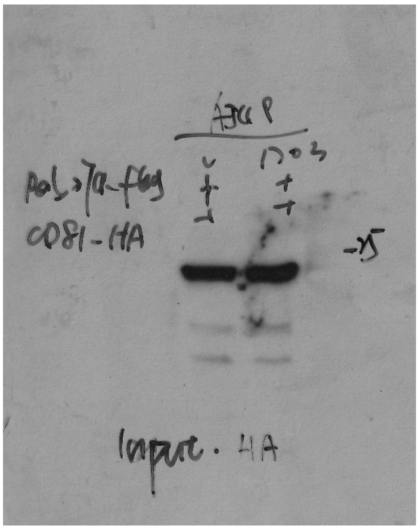

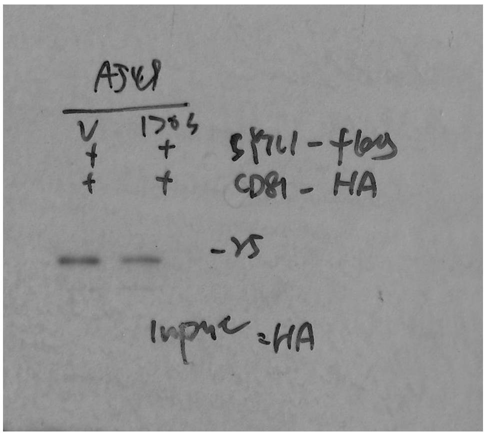

Figure S5

E

Repeat 1

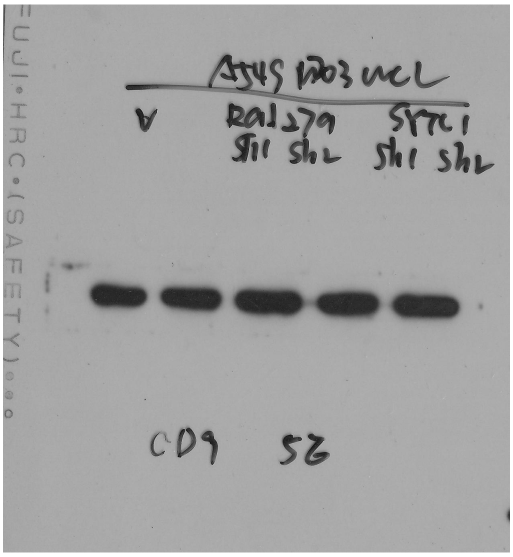

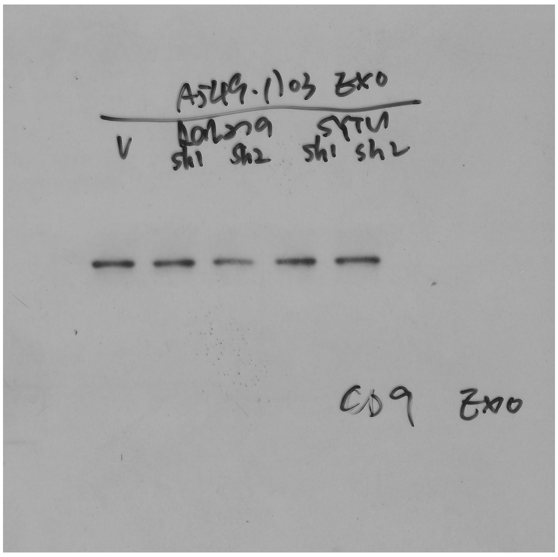

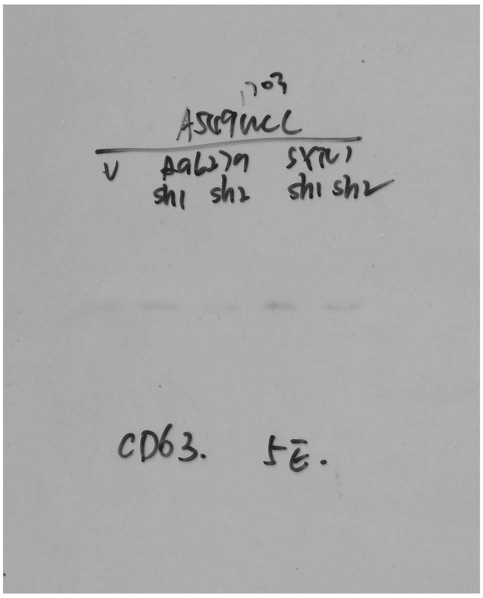

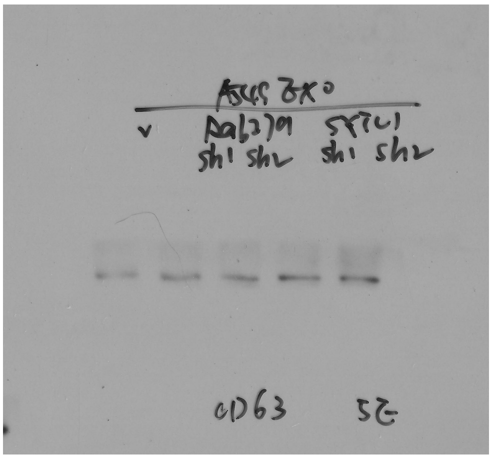

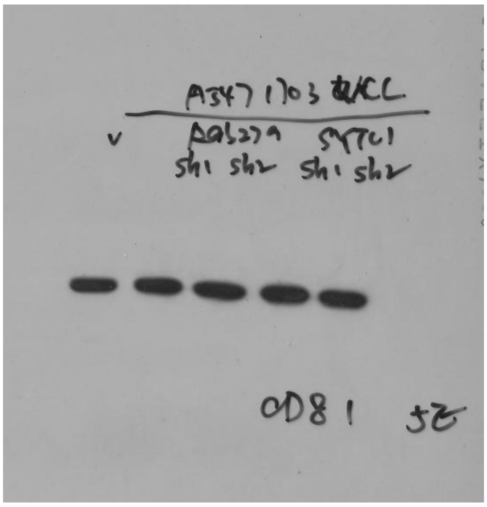

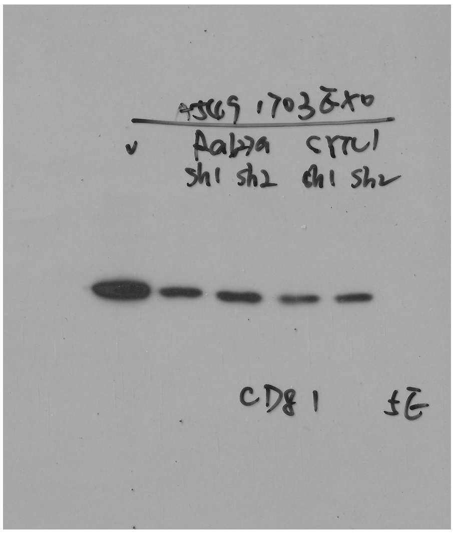

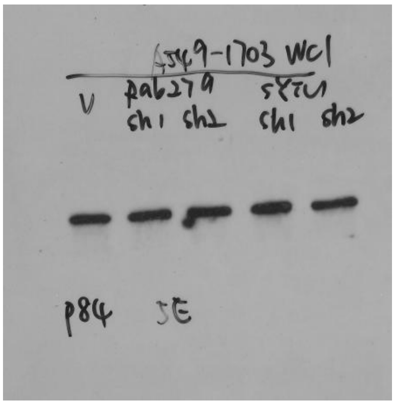

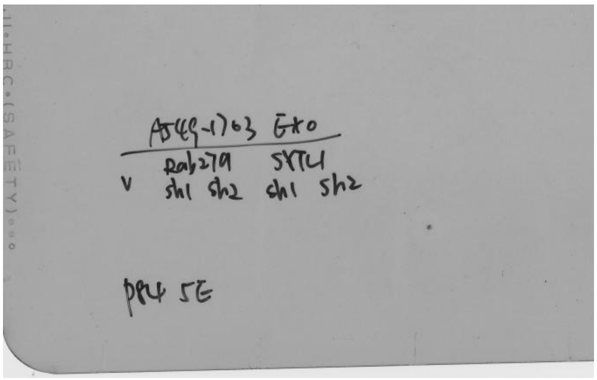

Repeat 2

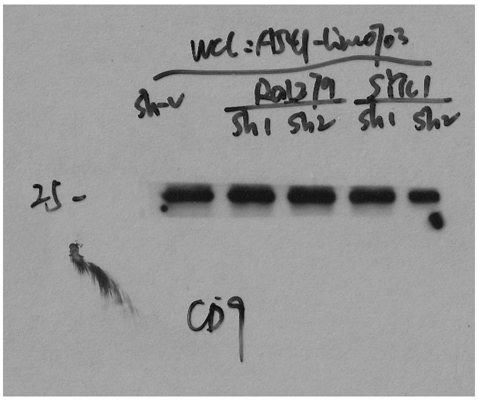

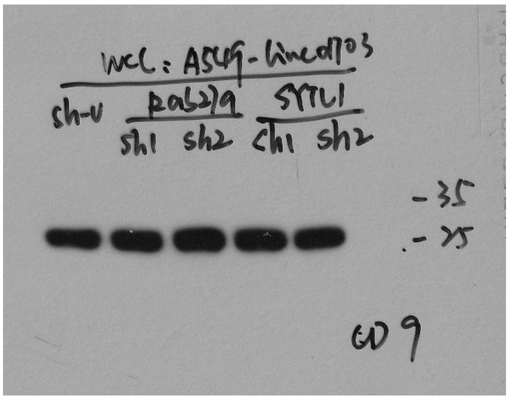

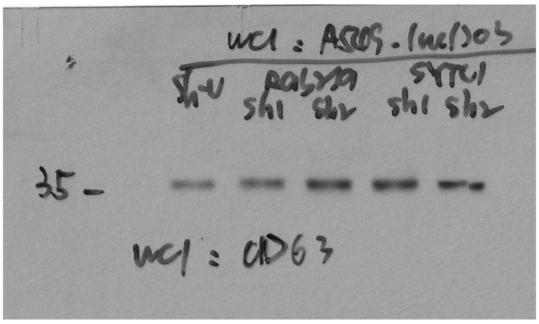

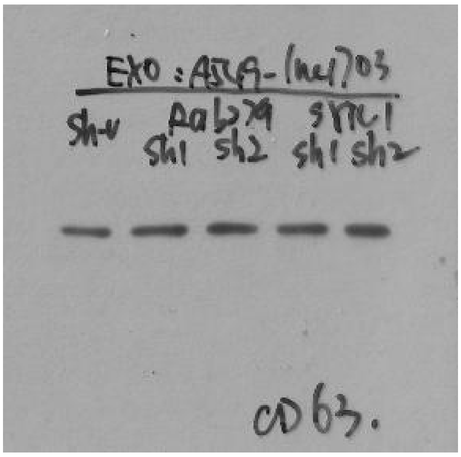

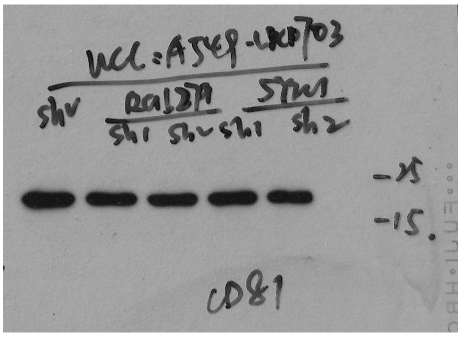
sh-V
Sh1 Sh2 Sh1 Sh2
-25
-15.
CD81

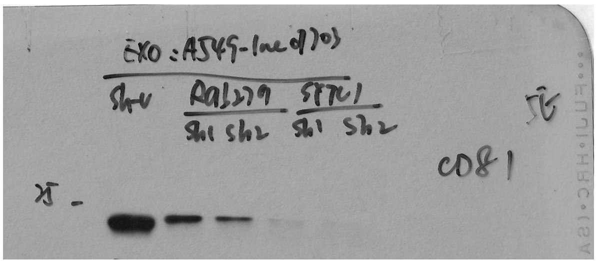
Sh-V
Sh1 Sh2 Sh1 Sh2
CD81
25 -

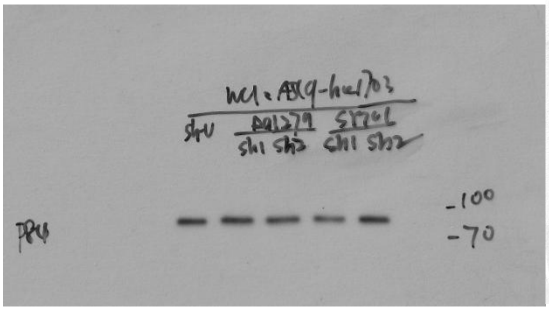
sh-V
sh1 sh2 sh1 sh2
-100
-70

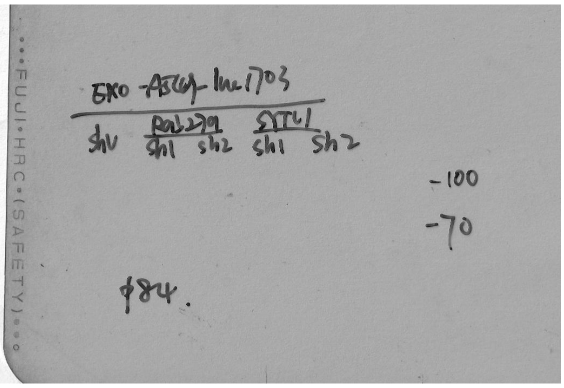
sh-V
sh1 sh2 sh1 sh2
-100
-70

Repeat 3

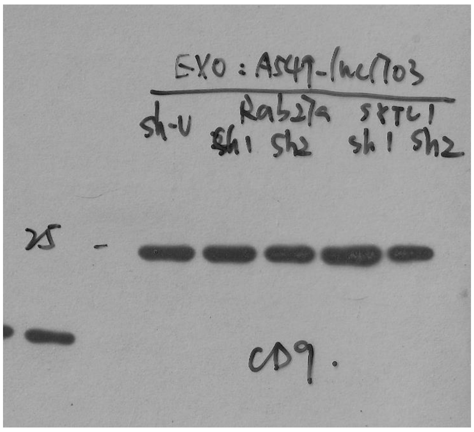
sh-V
sh1 sh2 sh1 sh2
25 -
CD9.

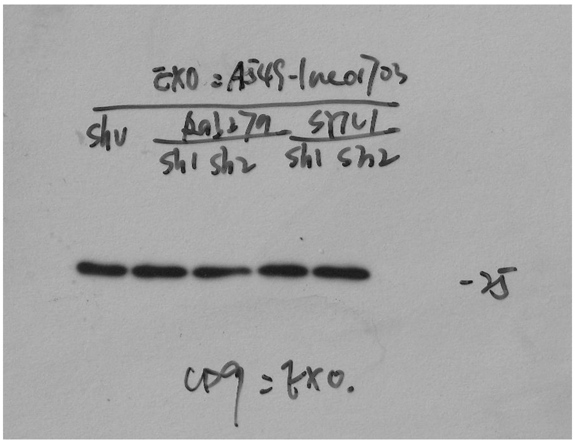
sh-V
sh1 sh2 sh1 sh2
-25

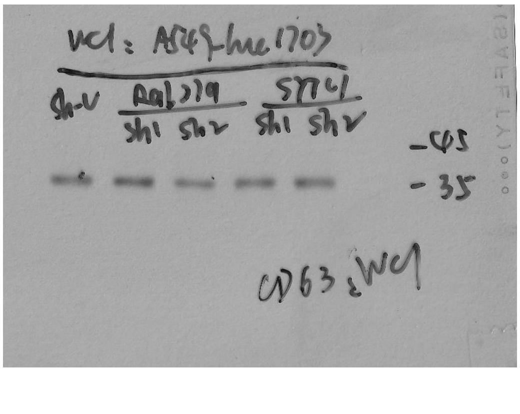
sh-V
sh1 sh2 sh1 sh2
-45
-35

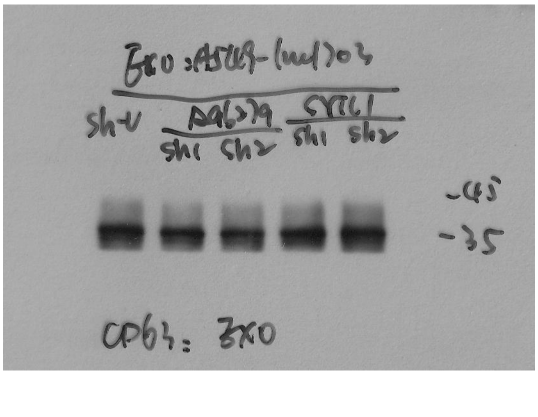
sh-V
sh1 sh2 sh1 sh2
-45
-35

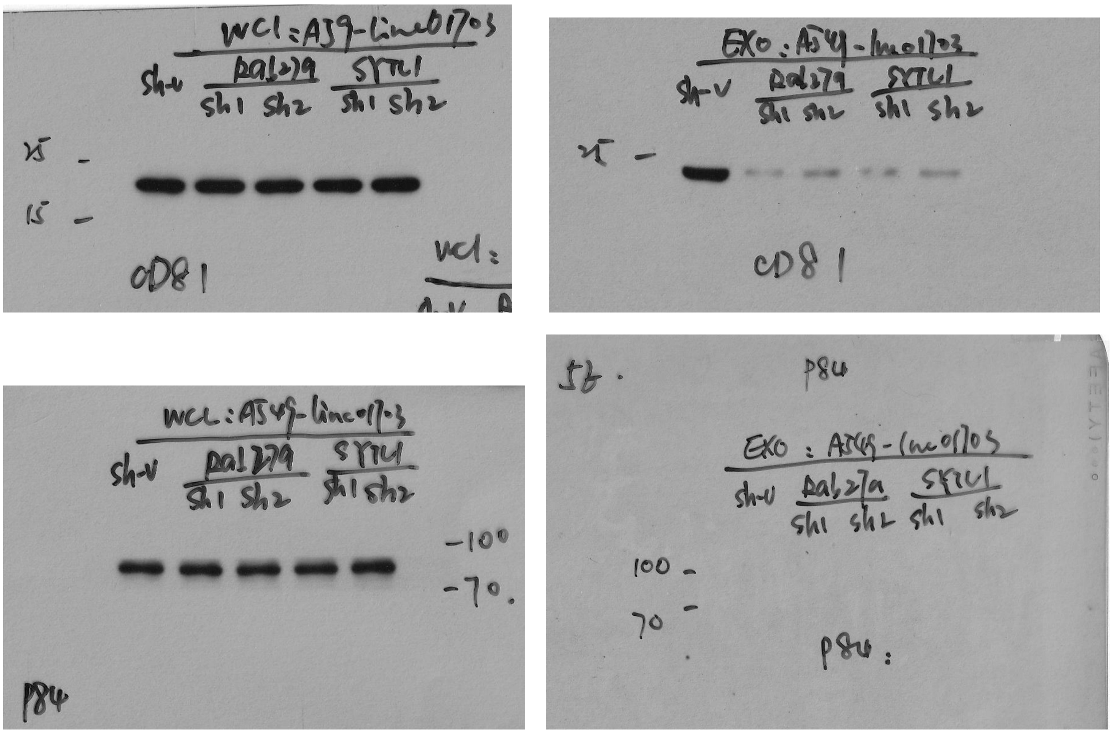

Figure S5

G

Repeat 1

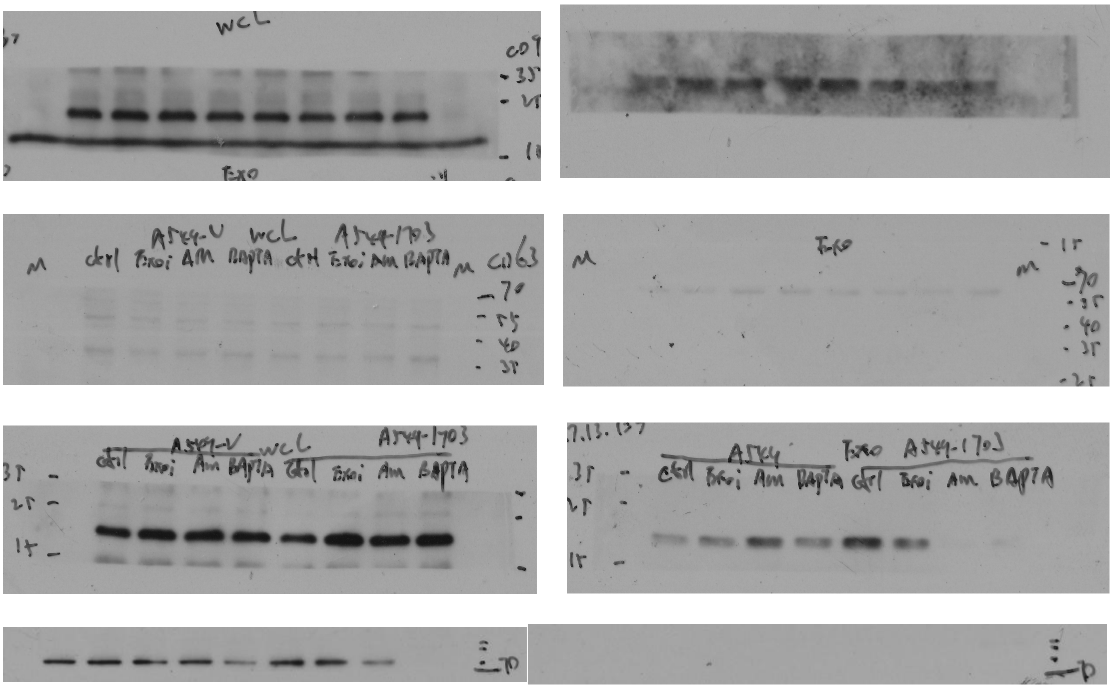

Repeat 2

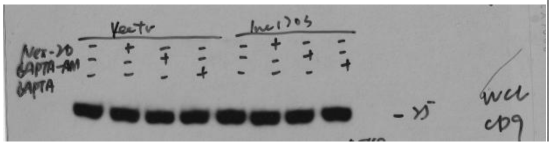

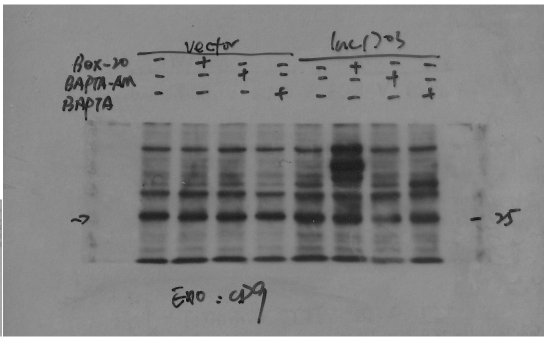

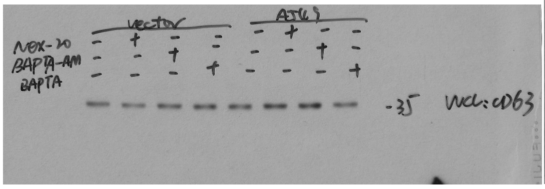

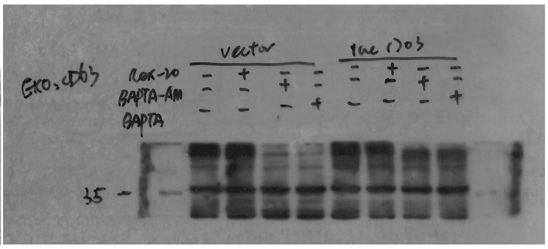

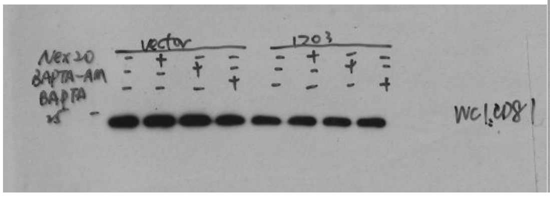

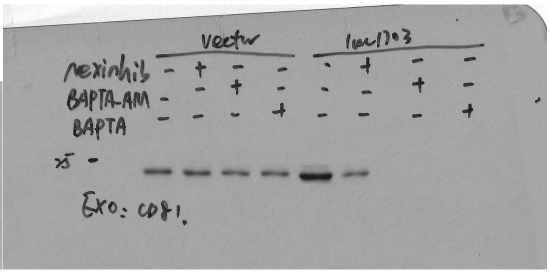

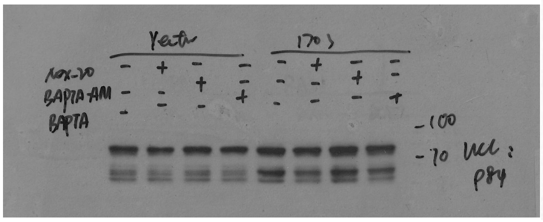

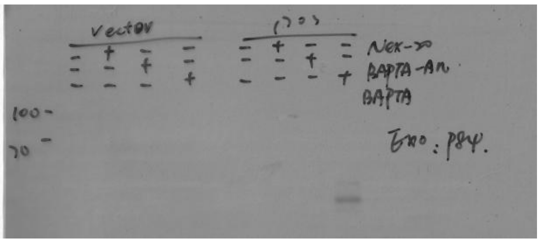

Repeat 3

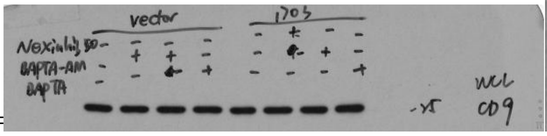

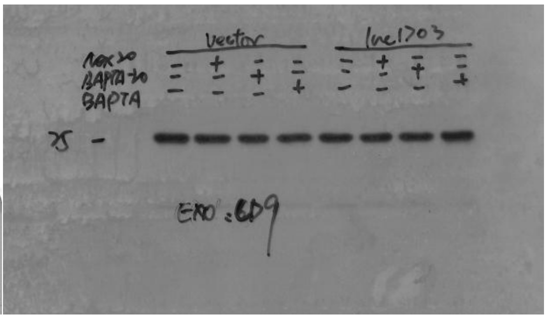

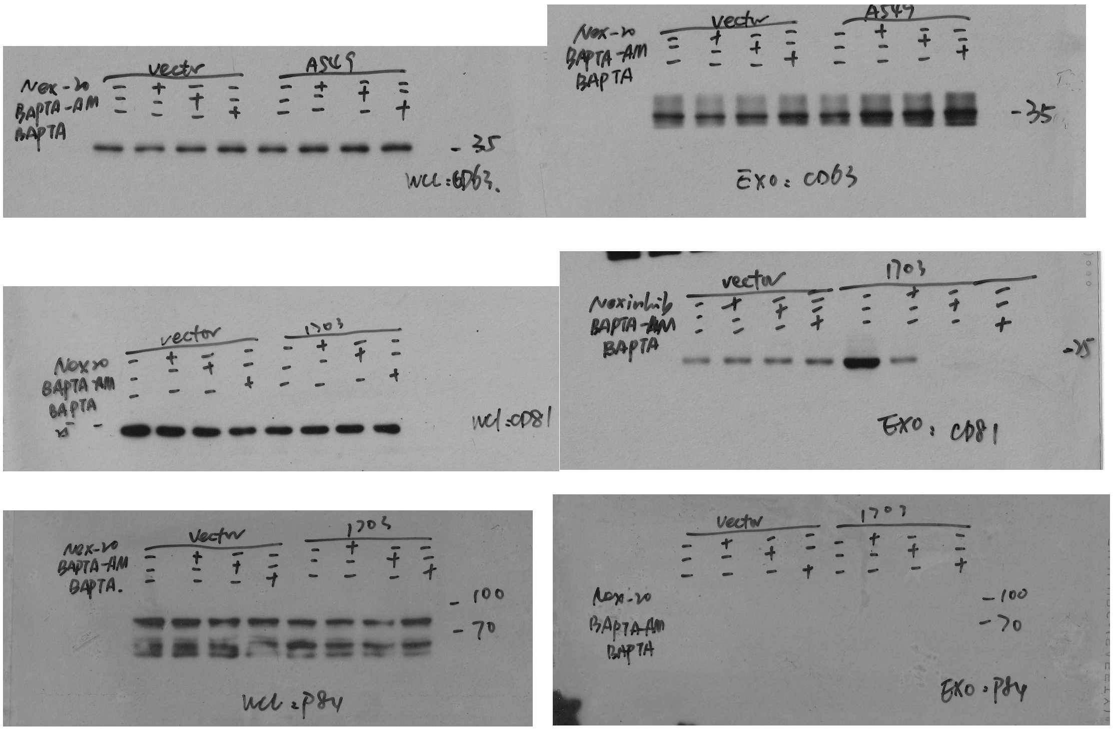
vector
A549
Nex-20
BAPTA-AM
BAPTA
-35
WCL: CD63
Exo: CD63
1703
Noxinhib
WCL: CD81
Exo: CD81
-25
-100
-70
WCL: P84
Exo: P84

Figure S6

C

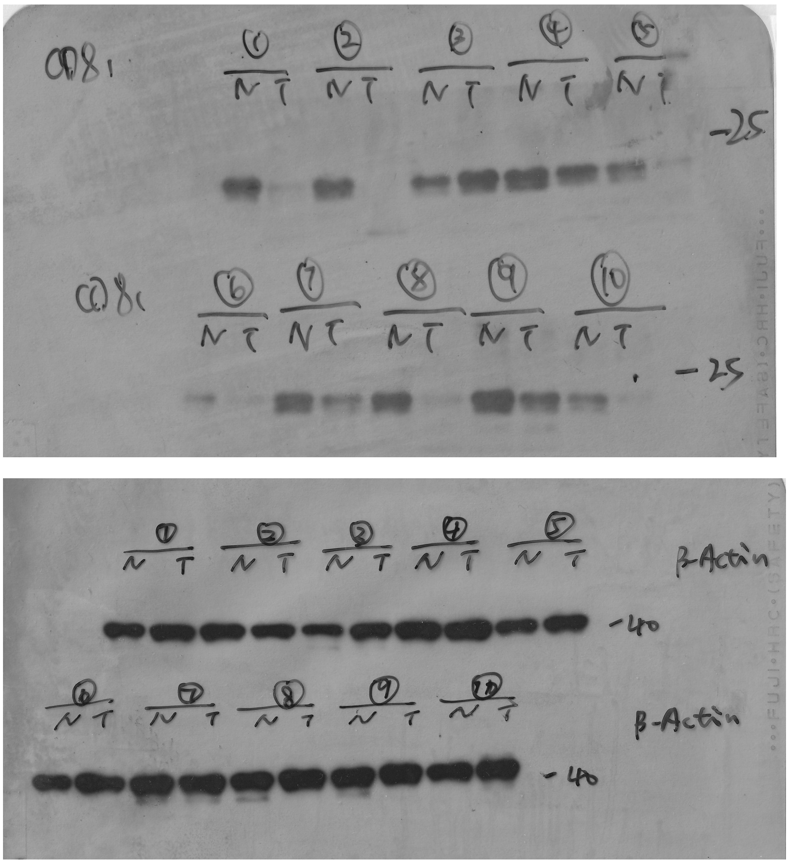
CD81
N T
-25
β-Actin
-40
