# Supplementary material for: LincRNA01703 Facilitates CD81+ Exosome Secretion to Inhibit Lung Adenocarcinoma Metastasis via the Rab27a/SYTL1/CD81 Complex
Source: Cancers (Basel). 2023 Dec 9;15(24):5781. doi: 10.3390/cancers15245781 (PMC10742068; doi:10.3390/cancers15245781)
Supplement: Supplementary file 1 [file cancers-15-05781-s001.zip › Supplementary Tables S1 and S2.pdf]

Supplementary Table S1：Oligos used for knockdown or knockout genes

CD81 sg1	CACCGTGATGACGCCAACAACGCCA
CD81 sg2	CACCGGGTGAAGACCTTCCACGAGA
Rab27a sh1	CCAGTGTACTTTACCAATATA
Rab27a sh2	CGGATCAGTTAAGTGAAGAAA
SYTL1 sh1	CCCTGTGTTCAATCACACCAT
SYTL1 sh2	GCGTCCCAGATCCTGGAGAAT

Supplementary Table S2: Sense and antisense primers used for qRT-PCR

Linc01703 sence:	ATACCCTAGGAGATGGAGGAAC
Linc01703 antisence:	GGCAACAGTGGCTTGAGATA
SYTL1 sence:	CCATCGCAAGAGGGGCTTT
SYTL1 antisence:	TCCAACAGTCCTTCAGTCTCA
SYTL2 sence:	GCCCAGTGTAAGGACTTAGCA
SYTL2 antisence:	GCCTTTGTCTGGTAGCAAATAGG
SYTL3 sence:	GAGACCAGGCGGTTCAAAACA
SYTL3 antisence:	CCTTTCCACCGGAGATGCTG
SYTL4 sence:	CAACACTACAGTGATCGGACC
SYTL4 antisence:	ACAACCCCGACAAGTATTGGT
SYTL5 sence:	ATGATCCTGGGCGTCCTAAAG
SYTL5 antisence:	TCCCACTTCTACGTTTTGCTTC
MEIS1 sence:	GGGCATGGATGGAGTAGGC
MEIS1 antisence:	GGGTACTGATGCGAGTGCAG
PDCD6 sence:	ATGGCCGCCTACTCTTACC
PDCD6 antisence:	TCCTGTCTTTATCGACCCTCTG
Rab27A sence:	GCTTTGGGAGACTCTGGTGTA
Rab27A antisence:	TCAATGCCCACTGTTGTGATAAA
Rab27B sence:	TAGACTTTCGGGAAAAACGTGTG
Rab27B antisence:	AGAAGCTCTGTTGACTGGTGA

Rab35 sence:	TACTGTTGCGTTTTGCAGACA
Rab35 antisence:	CCCCGATAATACGTGGAGGTG
U6 sence:	CTCGCTTCGGCAGCACA
U6 antisence:	AACGCTTCACGAATTTGCGT
β-Actin sence:	CATGTACGTTGCTATCCAGGC
β-Actin antisence:	CTCCTTAATGTCACGCACGAT
GAPDH sence:	GGAGCGAGATCCCTCCAAAAT
GAPDH antisence:	GGCTGTTGTCATACTTCTCATGG
